# Supplementary material for: Efficient precise in vivo base editing in adult dystrophic mice
Source: Nat Commun. 2021 Jun 17;12:3719. doi: 10.1038/s41467-021-23996-y (PMC8211797; doi:10.1038/s41467-021-23996-y)
Supplement: Supplementary file 1 — Supplementary Information [file 41467_2021_23996_MOESM1_ESM.pdf]

Efficient precise *in vivo* base editing in adult dystrophic mice

First author's surname: Xu

Short title: *in vivo* base editing of DMD mice

Li Xu[1†], Chen Zhang[1†], Haiwen Li[1], Peipei Wang[1], Yandi Gao[1], Nahush A. Mokadam[1], Jianjie Ma[1], William. D. Arnold[2], Renzhi. Han[1*]

Supplemental Material

Supplementary Table S1. List of pathogenic G:C to A:T point mutations in DMD.

chrom	pos	ref	alt	hgvs_c	variant_type
X	31206668	C	T	NM_004006.2:c.9564-1G>A	splice acceptor variant
X	32491387	G	A	NM_004006.2:c.2512C>T	nonsense
X	32217062	G	A	NM_004006.2:c.6292C>T	nonsense
X	32644314	C	T	NM_004006.2:c.1150-1G>A	splice acceptor variant
X	31177932	G	A	NM_004006.2:c.10262C>T	500B downstream variant
X	31496871	G	A	NM_004006.2:c.8464C>T	nonsense
X	32645052	C	T	NM_004006.2:c.1061G>A	nonsense
X	32491414	G	A	NM_004006.2:c.2485C>T	nonsense
X	31169519	G	A	NM_004020.3:c.2843+9150C	intron variant
X	32365175	G	A	NM_004006.2:c.4870C>T	nonsense
X	31178784	G	A	NM_004006.2:c.10108C>T	nonsense
X	32342123	G	A	NM_004006.2:c.5899C>T	nonsense
X	32438240	C	T	NM_004006.2:c.4071+1G>A	splice donor variant
X	31729736	C	T	NM_004006.2:c.7555G>A	missense variant
X	33174335	C	T	NM_004006.2:c.31+36947G>	intron variant
X	31658123	G	A	NM_004006.2:c.7894C>T	nonsense
X	32472247	G	A	NM_004006.2:c.2866C>T	nonsense
X	32438372	G	A	NM_004006.2:c.3940C>T	nonsense
X	32364704	G	A	NM_004006.2:c.5032C>T	nonsense
X	31729634	G	A	NM_004006.2:c.7657C>T	nonsense
X	31507280	C	T	NM_004006.2:c.8390+1G>A	splice donor variant
X	32823316	C	T	NM_004006.2:c.336G>A	5 prime UTR variant
X	32343232	G	A	NM_004006.2:c.5641C>T	nonsense
X	32844794	G	A	NM_004006.2:c.253C>T	5 prime UTR variant
X	32468539	G	A	NM_004006.2:c.3121C>T	nonsense
X	31875331	G	A	NM_004006.2:c.6955C>T	5 prime UTR variant
X	32849736	G	A	NM_004006.2:c.178C>T	5 prime UTR variant
X	32390103	G	A	NM_004006.2:c.4312C>T	nonsense
X	32342105	G	A	NM_004006.2:c.5917C>T	nonsense
X	32501833	G	A	NM_004006.2:c.2302C>T	nonsense
X	31173588	G	A	NM_004020.3:c.2843+5081C	intron variant
X	32464674	C	T	NM_004006.2:c.3188G>A	nonsense
X	32217037	C	T	NM_004006.2:c.6317G>A	nonsense
X	32362826	G	A	NM_004006.2:c.5287C>T	nonsense
X	32573766	C	T	NM_004006.2:c.1683G>A	nonsense
X	31180423	G	A	NM_004006.2:c.10033C>T	nonsense
X	32595756	C	T	NM_004006.2:c.1602+1G>A	splice donor variant
X	31479043	G	A	NM_004006.2:c.8608C>T	nonsense
X	32697947	G	A	NM_004006.2:c.883C>T	nonsense

X	31679565	C	T	NM_004006.2:c.7682G>A	nonsense
X	32816541	G	A	NM_004006.2:c.457C>T	nonsense
X	32348501	G	A	NM_004006.2:c.5353C>T	nonsense
X	32411811	G	A	NM_004006.2:c.4174C>T	nonsense
X	31180369	C	T	NM_004006.2:c.10086+1G>A	splice donor variant
X	32573529	C	T	NM_004006.2:c.1812+1G>A	splice donor variant
X	32389536	G	A	NM_004006.2:c.4483C>T	nonsense
X	32573812	C	T	NM_004006.2:c.1637G>A	nonsense
X	32364647	G	A	NM_004006.2:c.5089C>T	nonsense
X	32342154	C	T	NM_004006.2:c.5868G>A	nonsense
X	32518098	C	T	NM_004006.2:c.2202G>A	nonsense
X	32472252	C	T	NM_004006.2:c.2861G>A	nonsense
X	32485072	G	A	NM_004006.2:c.2650C>T	nonsense
X	31223071	G	A	NM_004006.2:c.9337C>T	nonsense
X	32595765	G	A	NM_004006.2:c.1594C>T	nonsense
X	32573834	G	A	NM_004006.2:c.1615C>T	nonsense
X	32485057	G	A	NM_004006.2:c.2665C>T	nonsense
X	32849781	G	A	NM_004006.2:c.133C>T	5 prime UTR variant
X	32501767	G	A	NM_004006.2:c.2368C>T	nonsense
X	32545250	G	A	NM_004006.2:c.2077C>T	nonsense
X	32816565	G	A	NM_004006.2:c.433C>T	nonsense
X	31774193	C	T	NM_004006.2:c.7310-1G>A	splice acceptor variant
X	32699111	C	T	NM_004006.2:c.831+1G>A	splice donor variant
X	31679492	C	T	NM_004006.2:c.7755G>A	nonsense
X	32699219	G	A	NM_004006.2:c.724C>T	nonsense
X	32491480	G	A	NM_004006.2:c.2419C>T	nonsense
X	32411772	G	A	NM_004006.2:c.4213C>T	nonsense
X	32468683	G	A	NM_004006.2:c.2977C>T	nonsense
X	32448495	C	T	NM_004006.2:c.3747G>A	nonsense
X	31496892	G	A	NM_004006.2:c.8443C>T	nonsense
X	32411868	G	A	NM_004006.2:c.4117C>T	nonsense
X	32565704	G	A	NM_004006.2:c.1990C>T	nonsense
X	31627852	G	A	NM_004006.2:c.8038C>T	nonsense
X	32345999	G	A	NM_004006.2:c.5530C>T	nonsense
X	32468573	C	T	NM_004006.2:c.3087G>A	nonsense
X	32809492	C	T	NM_004006.2:c.649+1G>A	splice donor variant
X	31820055	C	T	NM_004006.2:c.7229G>A	5 prime UTR variant
X	32816509	C	T	NM_004006.2:c.489G>A	nonsense
X	31206663	G	A	NM_004006.2:c.9568C>T	nonsense
X	31478129	G	A	NM_004006.2:c.8914C>T	nonsense
X	32614320	G	A	NM_004006.2:c.1465C>T	nonsense

X	32595855	G	A	NM_004006.2:c.1504C>T	nonsense
X	33211304	C	T	NM_004006.2:c.9G>A	nonsense
X	31223046	C	T	NM_004006.2:c.9361+1G>A	splice donor variant
X	32545158	C	T	NM_004006.2:c.2168+1G>A	splice donor variant
X	31178668	C	T	NM_004006.2:c.10223+1G>A	synonymous variant
X	32216981	G	A	NM_004006.2:c.6373C>T	nonsense
X	32454833	C	T	NM_004006.2:c.3433-1G>A	splice acceptor variant
X	32573744	C	T	NM_004006.2:c.1704+1G>A	splice donor variant
X	32389614	G	A	NM_004006.2:c.4405C>T	nonsense
X	32650983	G	A	NM_004006.2:c.961-5831C>T	intron variant
X	32491492	G	A	NM_004006.2:c.2407C>T	nonsense
X	32390175	G	A	NM_004006.2:c.4240C>T	nonsense
X	31478983	C	T	NM_004006.2:c.8668G>A	missense variant
X	31182784	G	A	NM_004006.2:c.9928C>T	nonsense
X	32545310	G	A	NM_004006.2:c.2017C>T	nonsense
X	31478995	G	A	NM_004006.2:c.8656C>T	nonsense
X	32454685	G	A	NM_004006.2:c.3580C>T	nonsense
X	32809559	G	A	NM_004006.2:c.583C>T	nonsense
X	32484925	G	A	NM_004006.2:c.2797C>T	nonsense
X	32545295	G	A	NM_004006.2:c.2032C>T	nonsense
X	32573786	G	A	NM_004006.2:c.1663C>T	nonsense
X	31507313	C	T	NM_004006.2:c.8358G>A	nonsense
X	32491463	C	T	NM_004006.2:c.2436G>A	nonsense
X	31658118	C	T	NM_004006.2:c.7899G>A	nonsense
X	31348571	G	A	NM_004006.2:c.9148C>T	nonsense
X	32463444	G	A	NM_004006.2:c.3427C>T	nonsense
X	31180437	C	T	NM_004006.2:c.10019G>A	missense variant
X	32644131	C	T	NM_004006.2:c.1331+1G>A	splice donor variant
X	32614397	C	T	NM_004006.2:c.1388G>A	nonsense
X	32573796	C	T	NM_004006.2:c.1653G>A	nonsense
X	32849727	C	T	NM_004006.2:c.186+1G>A	splice donor variant
X	32565742	C	T	NM_004006.2:c.1952G>A	nonsense
X	32491344	C	T	NM_004006.2:c.2555G>A	nonsense
X	32491276	C	T	NM_004006.2:c.2622+1G>A	splice donor variant
X	32484918	C	T	NM_004006.2:c.2803+1G>A	splice donor variant
X	32472310	C	T	NM_004006.2:c.2804-1G>A	splice acceptor variant
X	32464585	C	T	NM_004006.2:c.3276+1G>A	splice donor variant
X	32463458	C	T	NM_004006.2:c.3413G>A	nonsense
X	32463438	C	T	NM_004006.2:c.3432+1G>A	splice donor variant
X	32816641	C	T	NM_004006.2:c.358-1G>A	splice acceptor variant
X	32389496	C	T	NM_004006.2:c.4518+5G>A	intron variant

X	32287702	C	T	NM_004006.2:c.6118-1G>A	splice acceptor variant
X	31929602	C	T	NM_004006.2:c.6906G>A	5 prime UTR variant
X	31819974	C	T	NM_004006.2:c.7309+1G>A	splice donor variant
X	31679564	C	T	NM_004006.2:c.7683G>A	nonsense
X	31679430	C	T	NM_004006.2:c.7817G>A	nonsense
X	31679429	C	T	NM_004006.2:c.7818G>A	nonsense
X	31507314	C	T	NM_004006.2:c.8357G>A	nonsense
X	31478163	C	T	NM_004006.2:c.8880G>A	nonsense
X	33020138	C	T	NM_004006.2:c.93+1G>A	splice donor variant
X	32849821	C	T	NM_004006.2:c.94-1G>A	splice acceptor variant
X	31209497	C	T	NM_004006.2:c.9563+1G>A	splice donor variant
X	31206581	C	T	NM_004006.2:c.9649+1G>A	splice donor variant
X	31182861	C	T	NM_004006.2:c.9851G>A	nonsense
X	31178751	G	A	NM_004006.2:c.10141C>T	nonsense
X	31178721	G	A	NM_004006.2:c.10171C>T	nonsense
X	31178700	G	A	NM_004006.2:c.10192C>T	nonsense
X	31147421	G	A	NM_004006.2:c.10651C>T	nonsense
X	32645020	G	A	NM_004006.2:c.1093C>T	nonsense
X	32644202	G	A	NM_004006.2:c.1261C>T	nonsense
X	32644139	G	A	NM_004006.2:c.1324C>T	nonsense
X	32595870	G	A	NM_004006.2:c.1489C>T	nonsense
X	32565782	G	A	NM_004006.2:c.1912C>T	nonsense
X	32545190	G	A	NM_004006.2:c.2137C>T	nonsense
X	32501803	G	A	NM_004006.2:c.2332C>T	nonsense
X	32484964	G	A	NM_004006.2:c.2758C>T	nonsense
X	32468704	G	A	NM_004006.2:c.2956C>T	nonsense
X	32468509	G	A	NM_004006.2:c.3151C>T	nonsense
X	32464603	G	A	NM_004006.2:c.3259C>T	nonsense
X	32463576	G	A	NM_004006.2:c.3295C>T	nonsense
X	32454778	G	A	NM_004006.2:c.3487C>T	nonsense
X	32823297	G	A	NM_004006.2:c.355C>T	5 prime UTR variant
X	32389644	G	A	NM_004006.2:c.4375C>T	nonsense
X	32389605	G	A	NM_004006.2:c.4414C>T	nonsense
X	32380626	G	A	NM_004006.2:c.4729C>T	nonsense
X	32365049	G	A	NM_004006.2:c.4996C>T	nonsense
X	32364605	G	A	NM_004006.2:c.5131C>T	nonsense
X	32364602	G	A	NM_004006.2:c.5134C>T	nonsense
X	32346023	G	A	NM_004006.2:c.5506C>T	nonsense
X	32345978	G	A	NM_004006.2:c.5551C>T	nonsense
X	32345975	G	A	NM_004006.2:c.5554C>T	nonsense
X	32809577	G	A	NM_004006.2:c.565C>T	nonsense

X	32342264	G	A	NM_004006.2:c.5758C>T	nonsense
X	32342171	G	A	NM_004006.2:c.5851C>T	nonsense
X	32287680	G	A	NM_004006.2:c.6139C>T	nonsense
X	32287596	G	A	NM_004006.2:c.6223C>T	nonsense
X	32287536	G	A	NM_004006.2:c.6283C>T	nonsense
X	31929718	G	A	NM_004006.2:c.6790C>T	5 prime UTR variant
X	31836729	G	A	NM_004006.2:c.7189C>T	5 prime UTR variant
X	31679575	G	A	NM_004006.2:c.7672C>T	nonsense
X	31627681	G	A	NM_004006.2:c.8209C>T	nonsense
X	31478330	G	A	NM_004006.2:c.8713C>T	nonsense
X	31444621	G	A	NM_004006.2:c.8944C>T	nonsense
X	31223062	G	A	NM_004006.2:c.9346C>T	nonsense

Supplementary Table S2. List of ABE variants engineered in this study.

Name	Description
ABE-NG	ABEmax with SpCas9-NG mutations R1335V/L1111R/D1135V/G1218R/E1219F/A1322R/T1337R
ABE-NGA	ABE-NG with R1335Q mutation
ABE-NGC	ABE-NG with R1335E mutation
ABE-NG-loop	ABE-NG with the loop sequence from ScCas9 (amino acids 367-376) inserted
ABE-NGX	ABE-NG with A262T/R324L/S409I/E480K/E543D/M694I mutations
ABE-NGX-NGA	ABE-NGX with R1335Q
ABE-NGX-NGC	ABE-NGX with R1335E
ABE-NGX-loop	ABE-NGX with the loop sequence from ScCas9 (amino acids 367-376) inserted
ABEmaxSc	ABEmax with SpCas9 nickase replaced with ScCas9 nickase
ABE-NGm	ABE-NG with the dimeric TadA-TadA* replaced with monomeric TadA* containing two additional mutations A56G and V82G
iABE-NGA	ABE-NGA with the dimeric TadA-TadA* replaced with monomeric TadA* containing two additional mutations A56G and V82G

Supplementary Table S3. List of off-target DNA editing sites predicted by CRISPOR.

# Sequence	ACAGCTGCAGAACAGGAGATAAC
# Genome	mm10
# PAM	NGN
# Position	?
# Version	CRISPOR 4.98, 2020-12-14T17:04:41CET
# Results	http://crispor.org/crispor.py?batchId=CQzhVWcjhnN1VHP7yKi4

Name	offtargetSeq	mismatchPos	mismatchCount	chrom
OT1	ATTTT**A**TGCTGTTCTGCAGCTGG	*..*.*.*............	4	chr13
OT2	TTTG**A**CTTCTGTTCTGCAGCTGG	*..**..*............	4	chr5
OT3	CTTC**AA**TCCTGTTCTGCAGCAGG	*..***..............	4	chr3
OT4	GG**AA**GCCCCTGTTCTGCAGCAGA	.**.*.*.............	4	chr8
OT5	GCTCTGGCCTGTTCTGCAGCTGA	.*.*.**.............	4	chr2
OT6	CCTCCCTCCTGTTCTGCAGCTGC	**.**...............	4	chr5
OT7	GTGTCCTGCTGTTCTGCAGCAGT	..***..*............	4	chr1
OT8	CCTCTGTCCTGTTCTGCAGCTGT	**.*.*..............	4	chr6
OT9	ATT**A**GCT**A**CTGTTCTGCAGCCGG	*...*..*............	3	chr5
OT10	TTTCTTTCCTGTTCTGCAGCAGA	*..*.*..............	3	chr11
OT11	GTTGTCTCCTGTTCTGCAGCTGT	...*................	1	chrX
OT12	TTTAGCTTCTGCTCTGCAGCTGG	*...*..*...*........	4	chr12
OT13	GTTTCATCTTGTTCTGCAGCTGG	...***..*...........	4	chr6
OT14	ATTTTCTTCTATTCTGCAGCAGG	*..*...*..*.........	4	chr9
OT15	GCTTTTTCCTGCTCTGCAGCTGG	.*.*.*.....*........	4	chr7
OT16	GTTACCCACTGCTCTGCAGCAGG	....*.**...*........	4	chr9
OT17	TTTGTCTACTGCTCTGCAGCAGG	*..*...*...*........	4	chr18
OT18	GCTATATTCTTTTCTGCAGCAGG	.*...*.*..*.........	4	chr16
OT19	GGTATGTATTGTTCTGCAGCAGG	.*...*.**...........	4	chr16
OT20	GGTCTATCATGTTCTGCAGCTGG	.*.*.*..*...........	4	chr6
OT21	CCTACCTCCTTTTCTGCAGCCGG	**..*.....*.........	4	chr3
OT22	GGTGACTCATGTTCTGCAGCAGG	.*.**...*...........	4	chr9
OT23	TTGACCTCCTCTTCTGCAGCAGG	*.*.*.....*.........	4	chr11
OT24	GTTTTGTTCTGTTTTGCAGCGGG	...*.*.*.....*......	4	chr13
OT25	GCCTTCTCCAGTTCTGCAGCAGG	.***.....*..........	4	chr19
OT26	CTTGCCTCCTGTTCTGCACCAGG	*..**.............*.	4	chr2
OT27	GTTCTCAACTGCTCTGCAGCAGG	...*..**...*........	4	chr19
OT28	CTTATTTTCTGTTCTGCAGTGGG	*....*.*...........*	4	chr14
OT29	CTTATTTTCTGTTCTGCAGTAGG	*....*.*...........*	4	chr12
OT30	AATATGTCCTGTTCTGCACCAGG	**...*............*.	4	chr9
OT31	GCTAGGTCCTGTTCAGCAGCAGG	.*..**........*.....	4	chr11
OT32	GTCTTCTGTTGTTCTGCAGCTGG	..**...**...........	4	chr8
OT33	GATGGCTCCTGTTCTGCACCTGG	.*.**.............*.	4	chr16
OT34	GATGTCTACTCTTCTGCAGCAGG	.*.*...*..*.........	4	chr6
OT35	GTGATAGCCTTTTCTGCAGCGGG	..*..**...*.........	4	chr8
OT36	GTTGTACCCTGTGCTGCAGCAGG	...*.**.....*.......	4	chr11
OT37	TTCATGTCCTTTTCTGCAGCTGG	*.*..*....*.........	4	chr7
OT38	ATTGTGTCCTGTGCTGCAGCTGG	*..*.*......*.......	4	chr8
OT39	GTTTCCTTCTGCTCTGCAGCTGA	...**..*...*........	4	chr17
OT40	GATTTATCCTGTTCTACAGCTGA	.*.*.*.........*....	4	chr11
OT41	CCTGTCTCCTGCTCTGCAGCTGA	**.*.......*........	4	chr10
OT42	CTTATCAGCTGATCTGCAGCAGA	*.....**...*........	4	chr2
OT43	GACATTTCCTGTTCTGGAGCTGG	.**..*..........*...	4	chr1

OT44	GTTTCCTTCTGGTCTGCAGCTGA	...**..*...*........	4	chr17
OT45	TTAAGCTCCTGTTCTGCTGCTGA	*.*.*............*..	4	chr15
OT46	CTTATTCCCTGTTCTGCAGAAGA	*....**............*	4	chr10
OT47	GTTCTCCTTTGTTCTGCAGCTGA	...*..***...........	4	chr17
OT48	TTCATCTGCTGTTCTGCATCAGA	*.*....*..........*.	4	chr15
OT49	CTCATCTGCAGTTCTGCAGCAGA	*.*....*.*..........	4	chr10
OT50	TTTGCCTCCTGTTTTGCAGCTGC	*..**........*......	4	chr16
OT51	ATGAGCTCCTGTTCTGCAGAGGA	*.*.*..............*	4	chr6
OT52	TTTCTTTCCTTTTCTGCAGCAGA	*..*.*....*.........	4	chr9
OT53	GTGACATCCTGTCCTGCAGCTGC	..*.**......*.......	4	chr2
OT54	GTGATGTACTGTACTGCAGCTGA	..*..*.*....*.......	4	chr14
OT55	GATCTCTTCTCTTCTGCAGCTGA	.*.*...*..*.........	4	chr9
OT56	TTTGTCTTCAGTTCTGCAGCTGT	*..*...*.*..........	4	chr13
OT57	GTTCTTTGCTGTTCTGCAGAGGA	...*.*.*...........*	4	chr12
OT58	GTTGTACCCTGTGCTGCAGCTGA	...*.**.....*.......	4	chr5
OT59	TTTTTCTTCTGTTCAGCAGCAGC	*..*...*......*.....	4	chr15
OT60	CTTATTTACTATTCTGCAGCTGT	*....*.*..*.........	4	chr6
OT61	TTCATCTTCTGATCTGCAGCCGC	*.*....*...*........	4	chr12
OT62	GGTGTCCCCTGTCCTGCAGCAGC	.*.*..*.....*.......	4	chr3
OT63	TTTCTCTGCTTTTCTGCAGCAGA	*..*...*..*.........	4	chr6
OT64	GTGATCATCTGTACTGCAGCAGC	..*...**....*.......	4	chr7
OT65	GTTGTACCTTGTTCTGCAGCTGT	...*.**.*...........	4	chr3
OT66	GTTGTACCTTGTTCTGCAGCTGT	...*.**.*...........	4	chr1
OT67	TTTCCCTCCTGCTCTGCAGCAGT	*..**......*........	4	chr5
OT68	GCTGTCCCTTGTTCTGCAGCCGT	.*.*..*.*...........	4	chr2
OT69	CGTGTCTCATGTTCTGCAGCAGT	**.*....*...........	4	chr3
OT70	ATTTTTTCCTCTTCTGCAGCTGC	*..*.*....*.........	4	chr5
OT71	TTTATTTGCTGTTCTGCAGAGGT	*....*.*...........*	4	chr10
OT72	GTACTATCATGTTCTGCAGCTGC	..**.*..*...........	4	chr15
OT73	TTTATTGCCTGTTCTGCAGAGGT	*....**............*	4	chr3
OT74	CTTATGTGCAGTTCTGCAGCAGC	*....*.*.*..........	4	chr9
OT75	ATTTCCTCCTGTTCTGAAGCTGT	*..**...........*...	4	chr5
OT76	ATTCTCTGCTGCTCTGCAGCCGC	*..*...*...*........	4	chr16
OT77	GGTTTCCCCGGTTCTGCAGCGGC	.*.*..*..*..........	4	chr17
OT78	GTGGTTTCCTGATCTGCAGCAGT	..**.*.....*........	4	chr17
OT79	GCTCTCTTGTGTTCTGCAGCAGC	.*.*...**...........	4	chr9
OT80	GCTCCCTCCCGTTCTGCAGCAGC	.*.**....*..........	4	chr12
OT81	CTCATATCCTGTTTTGCAGCTGT	*.*..*.......*......	4	chr11
OT82	TTGATCACCTGTTCTGAAGCTGT	*.*...*.........*...	4	chr6
OT83	TTTCTTTCCTGTTCTGCACCTGC	*..*.*............*.	4	chrX
OT84	CTTAGTTCCTGTTCTGTAGCTGT	*...**..........*...	4	chr2
OT85	ATTAGCTACTGTTCTGCTGCTGT	*...*..*.........*..	4	chr12
OT86	GTGACTTCCTGTGCTGCAGCTGC	..*.**......*.......	4	chr5
OT87	GCCATCCCCTGTTCTGAAGCTGC	.**...*.........*...	4	chr8
OT88	GCCAGCTCCTGTTCTGCTGCTGC	.**.*............*..	4	chr1
OT89	TTTAGCTACTTTTCTGCAGCTGT	*...*..*..*.........	4	chr1
OT90	GTTTAGTCCTATTCTGCAGCGGT	...***....*.........	4	chr19
OT91	GTTCTGTTCTGCTCTGCAGCAGT	...*.*.*...*........	4	chrX
OT92	GCTCTGTCCTGCTCTGCAGCTGT	.*.*.*.....*........	4	chr18
OT93	GTTGTGACCTGTTCTGCAGAAGT	...*.**............*	4	chr18
OT94	CTTCTCACCTCTTCTGCAGCTGC	*..*..*...*.........	4	chr12
OT95	GTTATGGACTGTTCAGCAGCTGT	.....***......*.....	4	chr16

OT96	TTCATCTACTTTTCTGCAGCAGT	*.*....*..*.........	4	chr16
OT97	TTGATGTCCTGTGCTGCAGCAGC	*.*..*......*.......	4	chr3
OT98	TTTCACTCCTGGTCTGCAGCTGT	*..**......*........	4	chr5
OT99	GCTATTTTCTGTTCTGCAGGTGC	.*...*.*...........*	4	chr9
OT100	GTGTTCTGCTGTTCTGGAGCTGA	..**...*........*...	4	chr19
OT101	TTTGTATCCTGTTCTGCAGGAGC	*..*.*.............*	4	chr15
OT102	TTGATCTGCTGTTCTGCAGGGGT	*.*....*...........*	4	chr6
OT103	GTGCTCACCTGTTCTGCAGGGGT	..**..*............*	4	chr6
OT104	GCTCTGTCCTGTTCTCCAGCTGT	.*.*.*.........*....	4	chr19
OT105	GGTTTATCCTGTTCTTCAGCAGG	.*.*.*.........*....	4	chr15
OT106	ATTAACCCCTGTTCTTCAGCAGC	*...*.*........*....	4	chr15
OT107	ATTATTTTCTGTTCTCCAGCAGT	*....*.*.......*....	4	chr5
OT108	TCTTTCTCCTGTTCTTCAGCTGG	**.*...........*....	4	chr1
OT109	GTTACTTTCTGTTCTCCAGCAGG	....**.*.......*....	4	chr18
OT110	ACTAACTCCTGTTCTCCAGCAGT	**..*..........*....	4	chr17
OT111	GCTATGTACTGTTCTTCAGCTGT	.*...*.*.......*....	4	chr19
OT112	TTTTTCACCTGTTCTTCAGCAGT	*..*..*........*....	4	chr3
OT113	GTTAACAACTGTTCTCCAGCTGT	....*.**.......*....	4	chr18
OT114	GCTAAATCCTGTTCTTCAGCTGA	.*..**.........*....	4	chr10
OT115	TCTTTCTCCTGTTCTTCAGCAGA	**.*...........*....	4	chr8
OT116	GTTTTGTCCTATTCTGCAGCTGG	...*.*....*.........	3	chr10
OT117	GATTTCTCCTGTTTTGCAGCCGA	.*.*.........*......	3	chr9
OT118	GGTCTCTCTTGTTCTGCAGCTGA	.*.*....*...........	3	chr13
OT119	GTACTCTCCTGTTCTGCAGGAGG	..**...............*	3	chr7
OT120	GTCCTCTCCTGTTCTGGAGCTGG	..**............*...	3	chr9
OT121	GTTGTATCTTGTTCTGCAGCTGT	...*.*..*...........	3	chr14
OT122	GGTACCTCCTCTTCTGCAGCTGC	.*..*.....*.........	3	chr8
OT123	GTAACCTCCTCTTCTGCAGCAGC	..*.*.....*.........	3	chr16
OT124	GGTATCTGCTGGTCTGCAGCGGC	.*.....*...*........	3	chr2
OT125	GGAATCTCCTGTTCTGAAGCAGT	.**.............*...	3	chr4
OT126	CTTTTCTCCTGTTCTGCAGATGT	*..*...............*	3	chr4
OT127	GCTCTCTCCTGTTTTGCAGCAGT	.*.*.........*......	3	chr8
OT128	GTTGTTTCCTGTTATGCAGCTGT	...*.*.......*......	3	chr4
OT129	GTAGTCTCCTGTTCTGCAGGAGA	..**...............*	3	chr11
OT130	GTTCCCTCCTGTTCTTCAGCAGG	...**..........*....	3	chr13
OT131	CTTCTCTCCTGTTCTTCAGCTGG	*..*...........*....	3	chr6
OT132	GATATCTCCTGTTCTGCAGGAGA	.*.................*	2	chr1
OT133	GTTTTCTCCTGTTCTGCAGGTGT	...*...............*	2	chr11
OT134	GTTATCTCCTGCTCTGCAGCAGA	...........*........	1	chr16
OT135	TTTTTCTCCTACTCTGCAGCTGG	*..*......**........	4	chr4
OT136	TCTATCTCCAGTACTGCAGCAGG	**.......*..*.......	4	chr15
OT137	CTTATCACCTGCTTTGCAGCTGG	*.....*....*.*......	4	chr6
OT138	GTTATACCTTGTACTGCAGCAGG	.....**.*...*.......	4	chr9
OT139	GATATCTTCTGTTTAGCAGCAGG	.*.....*.....**.....	4	chr14
OT140	TATATCTCCTGGTCAGCAGCGGG	**.........*..*.....	4	chr6
OT141	GTACTCTCCTACTCTGCAGCTGG	..**......**........	4	chr5
OT142	ATTATCTGCTGATCTGCTGCTGG	*......*...*.....*..	4	chrX
OT143	ATTTTCTCCTATTCTGCGGCAGG	*..*......*......*..	4	chr6
OT144	GTAAGCTCCTATTCTGCAGATGG	..*.*.....*........*	4	chr8
OT145	GCTTTCTCCTGTCCTGCAGAGGG	.*.*........*......*	4	chr9
OT146	TTTATGTCCAGTTCTGCAGAGGG	*....*...*.........*	4	chr14
OT147	TTTATGTCAGGTTCTGCAGCTGG	*....*..**..........	4	chr1

OT148	TTTGTCTCCTCTCCTGCAGCAGG	*..*......*.*.......	4	chr3
OT149	GTGGTCTCCAGTCCTGCAGCTGG	..**.....*..*.......	4	chr7
OT150	GTTAGCTACTATTCTGCTGCTGG	....*..*..*......*..	4	chr5
OT151	CTTATCTGCAGGTCTGCAGCAGG	*......*.*.*........	4	chr4
OT152	GTTGTGTCTTGTACTGCAGCTGG	...*.*..*...*.......	4	chr7
OT153	GCTAACTCAGGTTCTGCAGCGGG	.*..*...**..........	4	chr19
OT154	GTTATACCCAGTGCTGCAGCGGG	.....**..*..*.......	4	chrX
OT155	GTTGTATCTTGTGCTGCAGCTGG	...*.*..*...*.......	4	chrX
OT156	GTTGTATCTTGTGCTGCAGCTGG	...*.*..*...*.......	4	chr16
OT157	GTTGTATCTTGTGCTGCAGCTGG	...*.*..*...*.......	4	chr4
OT158	GTTGTATCTTGTGCTGCAGCTGG	...*.*..*...*.......	4	chr12
OT159	GTTGTATCTTGTGCTGCAGCTGG	...*.*..*...*.......	4	chr4
OT160	GCTCTCTCTTGTTCTGCAGAGGG	.*.*....*..........*	4	chr1
OT161	GATACCTCCTGTCCTGCCGCTGG	.*..*.......*....*..	4	chr10
OT162	GGTCTCTCTTGTTCTGTAGCTGG	.*.*....*.......*...	4	chr14
OT163	GGTATTTCCTGTGCTGCTGCTGG	.*...*......*....*..	4	chr6
OT164	GTTGTCCCTTGTGCTGCAGCTGG	...*..*.*...*.......	4	chr5
OT165	GTTTTCCCCTCTTCTGCTGCTGG	...*..*...*......*..	4	chr3
OT166	TTCATCTCCTGTACTGCAGTGGG	*.*.........*......*	4	chr18
OT167	CTTGTCTCCTTGTCTGCAGCTGG	*..*......**........	4	chr8
OT168	GTTATCAGCGGTTCTGCAGTGGG	......**.*.........*	4	chr2
OT169	GTTTTTTCCTGTTCTGTAGTTGG	...*.*..........*..*	4	chr13
OT170	GGTTTCTCCTGTGCAGCAGCAGG	.*.*........*.*.....	4	chr11
OT171	GTGTTCTCCTGGTCTGCAGACGG	..**.......*.......*	4	chr8
OT172	GTTTTCTGCTTTTCTGCTGCTGG	...*...*..*......*..	4	chr8
OT173	GTTCTCCCATGTGCTGCAGCAGG	...*..*.*...*.......	4	chrX
OT174	GTTGCCTCCTGTTCCGCAGTTGG	...**.........*....*	4	chr10
OT175	GTTTTCTGCTGTGCTGAAGCTGG	...*...*....*...*...	4	chr5
OT176	TTTCTCTCCTGTGCTGCTGCCGG	*..*........*....*..	4	chr1
OT177	GTTACATCCTGATCTGCAGGGGG	....**.....*.......*	4	chr2
OT178	TTTATCCCTAGTTCTGCAGCAGA	*.....*.**..........	4	chr13
OT179	GATATCCCATGTTCTACAGCTGA	.*....*.*......*....	4	chr15
OT180	CTTATCCCCAGCTCTGCAGCAGA	*.....*..*.*........	4	chr1
OT181	CTTATCCCCAGCTCTGCAGCAGA	*.....*..*.*........	4	chr4
OT182	TTCATCTCCAGCTCTGCAGCAGA	*.*......*.*........	4	chr3
OT183	TTCATCTCCAGCTCTGCAGCAGA	*.*......*.*........	4	chr7
OT184	TTCATCTCCAGCTCTGCAGCAGA	*.*......*.*........	4	chr16
OT185	TGTATCTCCAGTTCTGCACCAGA	**.......*........*.	4	chr2
OT186	GTGCTCTCCTGTGCTGAAGCTGG	..**........*...*...	4	chr5
OT187	TTCATCTCCAGATCTGCAGCAGA	*.*......*.*........	4	chr1
OT188	GATATATCCTGTTCTGCATAAGA	.*...*............**	4	chr7
OT189	GTGAGCTCCTGCTCTGCAGGAGG	..*.*......*.......*	4	chr9
OT190	CTCATCTCCAGCTCTGCAGCAGA	*.*......*.*........	4	chr2
OT191	CTCATCTCCAGCTCTGCAGCAGA	*.*......*.*........	4	chr4
OT192	GGTATGTCCTGATTTGCAGCTGA	.*...*.....*.*......	4	chr8
OT193	ATTGTCTCCAGTTCTGCAGAGGA	*..*.....*.........*	4	chr14
OT194	GTCATCCCCAGCTCTGCAGCAGA	..*...*..*.*........	4	chrY
OT195	GGTATCTGCTGCTCTGTAGCTGA	.*.....*...*....*...	4	chr8
OT196	GTTATGTACTGTTTTACAGCTGA	.....*.*.....*.*....	4	chr3
OT197	TTTACCTCCTGTTCTGATGCCGA	*...*...........**..	4	chr10
OT198	TTAATCTCCTTTTCTGCAGGTGG	*.*.......*........*	4	chr14
OT199	CTTATCTACTATTCTGCAGATGA	*......*..*........*	4	chr2

OT200	GTTTTTTCCTCTTCTGCAGGTGG	...*.*....*........*	4	chr3
OT201	GTTTTTTCCTCTTCTGCAGGTGG	...*.*....*........*	4	chrX
OT202	TTTATGTCCTGTTCTGGAGATGG	*....*..........*..*	4	chr10
OT203	ATTGTCTCCTGTTTTGCAGATGA	*..*.........*.....*	4	chr11
OT204	GTTATGTAATGTTCGGCAGCAGG	.....*.**.....*.....	4	chr2
OT205	GAAATCTCATTTTCTGCAGCAGA	.**.....*.*.........	4	chr6
OT206	CTTAGCTCCAGTTCTGTAGCAGA	*...*....*......*...	4	chr19
OT207	GTTCTCCCAAGTTCTGCAGCTGA	...*..*.**..........	4	chr9
OT208	GTTATATTTTATTCTGCAGCTGT	.....*.**.*.........	4	chr12
OT209	GTTTCCTCCTTTTCTGCATCAGA	...**.....*.......*.	4	chr5
OT210	GATATGTCCTTCTCTGCAGCAGA	.*...*....**........	4	chr1
OT211	GATATCACCTGCTCTGCATCTGC	.*....*....*......*.	4	chr8
OT212	GTGATATCCTTATCTGCAGCAGA	..*..*....**........	4	chr1
OT213	GCTATCACATGCTCTGCAGCTGT	.*....*.*..*........	4	chr18
OT214	CTCATCTCCTGCTCTGTAGCAGA	*.*........*....*...	4	chr9
OT215	GCTATCACCTGCTCTGCCGCTGA	.*....*....*.....*..	4	chr15
OT216	GCTATCTTCTTTTCTGAAGCAGA	.*.....*..*.....*...	4	chr5
OT217	ATTATCTACTATTCTGCAACTGC	*......*..*.......*.	4	chrX
OT218	GTTATTACCTGCTCTGCAGATGC	.....**....*.......*	4	chr10
OT219	GTCATCTTCTGTTCGGCACCTGG	..*....*......*...*.	4	chr14
OT220	GCCATCTCCTGTTTTGCACCTGA	.**..........*....*.	4	chr14
OT221	GTTACTTCCTCCTCTGCAGCTGC	....**....**........	4	chr14
OT222	GCTATCTTCTATTCTGCAGAGGC	.*.....*..*........*	4	chr12
OT223	GTTAACTTCTGTTCAGTAGCAGA	....*..*......*.*...	4	chr1
OT224	GTCATCCCCAGTTCTGTAGCAGA	..*...*..*......*...	4	chr8
OT225	ATTATCCCCTGTTCTGTGGCTGA	*.....*.........**..	4	chr7
OT226	GTTCTCTGCTATTCTGCTGCTGA	...*...*..*......*..	4	chr7
OT227	GTTCTCTGCTATTCTGCTGCTGA	...*...*..*......*..	4	chr7
OT228	GTTCTCTGCTATTCTGCTGCTGA	...*...*..*......*..	4	chr7
OT229	CTCATCTCCACTTCTGCAGCAGA	*.*......**.........	4	chr5
OT230	GTTAACTTCTGATCTACAGCAGC	....*..*...*...*....	4	chr4
OT231	GCTCTCTCCTGTCCTGAAGCTGA	.*.*........*...*...	4	chrY
OT232	GCTCTCTCCTGTCCTGAAGCTGA	.*.*........*...*...	4	chrY
OT233	GCTCTCTCCTGTCCTGAAGCTGA	.*.*........*...*...	4	chrY
OT234	GCTCTCTCCTGTCCTGAAGCTGA	.*.*........*...*...	4	chrY
OT235	GCTCTCTCCTGTCCTGAAGCTGA	.*.*........*...*...	4	chrY
OT236	GCTCTCTCCTGTCCTGAAGCTGA	.*.*........*...*...	4	chrY
OT237	GCTCTCTCCTGTCCTGAAGCTGA	.*.*........*...*...	4	chrY
OT238	GCTCTCTCCTGTCCTGAAGCTGA	.*.*........*...*...	4	chrY
OT239	GCTCTCTCCTGTCCTGAAGCTGA	.*.*........*...*...	4	chrY
OT240	GCTCTCTCCTGTCCTGAAGCTGA	.*.*........*...*...	4	chrY
OT241	GCTCTCTCCTGTCCTGAAGCTGA	.*.*........*...*...	4	chrY
OT242	GCTCTCTCCTGTCCTGAAGCTGA	.*.*........*...*...	4	chrY
OT243	GCTCTCTCCTGTCCTGAAGCTGA	.*.*........*...*...	4	chrY
OT244	GCTCTCTCCTGTCCTGAAGCTGA	.*.*........*...*...	4	chrY
OT245	GCTCTCTCCTGTCCTGAAGCTGA	.*.*........*...*...	4	chrY
OT246	GCTCTCTCCTGTCCTGAAGCTGA	.*.*........*...*...	4	chrY
OT247	GCTCTCTCCTGTCCTGAAGCTGA	.*.*........*...*...	4	chrY
OT248	GCTCTCTCCTGTCCTGAAGCTGA	.*.*........*...*...	4	chrY
OT249	GCTCTCTCCTGTCCTGAAGCTGA	.*.*........*...*...	4	chrY
OT250	GCTCTCTCCTGTCCTGAAGCTGA	.*.*........*...*...	4	chrY
OT251	GCTCTCTCCTGTCCTGAAGCTGA	.*.*........*...*...	4	chrY

OT252	GCTCTCTCCTGTCCTGAAGCTGA	.*.*........*...*...	4	chrY
OT253	GTGATCTGCTGTACTGCAGAGGA	..*....*....*......*	4	chr1
OT254	CTTGTCTCCCGTTCTGAAGCAGA	*..*.....*......*...	4	chr14
OT255	GCTATATCCTCCTCTGCAGCTGC	.*...*....**........	4	chr2
OT256	TTGATCTCCTGTTAAGCAGCAGA	*.*..........**.....	4	chr9
OT257	TTTATCTGCTGTTTTGCATCTGC	*......*.....*....*.	4	chr15
OT258	GTTGTCCCTTGTGCTGCAGCTGA	...*..*.*...*.......	4	chr4
OT259	CTTATCACCTGATCAGCAGCAGC	*.....*....*..*.....	4	chr10
OT260	GTTATGACTTGTTTTGCAGCAGC	.....**.*....*......	4	chr7
OT261	GTCATCCCCTCTTCTGCATCAGA	..*...*...*.......*.	4	chr13
OT262	GTTTACTCCAGCTCTGCAGCAGC	...**....*.*........	4	chr8
OT263	GTAATTTCATCTTCTGCAGCTGC	..*..*..*.*.........	4	chr13
OT264	GTGATGTCATGTTATGCAGCAGA	..*..*..*....*......	4	chr3
OT265	GGTATCTACTGTTCTGCACTGGA	.*.....*..........**	4	chr17
OT266	ATTCTCTCCTGCTTTGCAGCTGC	*..*.......*.*......	4	chr12
OT267	GCTAGCTCCTCTTCTGCAGTGGA	.*..*.....*........*	4	chr4
OT268	GTTACCTACTGTTCTGTAACTGC	....*..*........*.*.	4	chrX
OT269	GTTATTTTCTGTTTTGCAGAAGT	.....*.*.....*.....*	4	chr8
OT270	GTTAGTTCCTATTCTGCTGCTGT	....**....*......*..	4	chr3
OT271	GTTACCTGCTGCTATGCAGCTGC	....*..*...*.*......	4	chrX
OT272	GGTGTCTCCGGTCCTGCAGCTGC	.*.*.....*..*.......	4	chr16
OT273	GCTATTTCCTGTTCTGCAAATGT	.*...*............**	4	chr15
OT274	GCTATCTTGTCTTCTGCAGCAGC	.*.....**.*.........	4	chr11
OT275	GCTATGTCCTGATCAGCAGCAGC	.*...*.....*..*.....	4	chr3
OT276	GGTATCTGCTATTCTGCTGCTGT	.*.....*..*......*..	4	chr3
OT277	GTGAGCTCTTGCTCTGCAGCCGT	..*.*...*..*........	4	chr5
OT278	GTTCTCTTTTGTACTGCAGCAGC	...*...**...*.......	4	chr5
OT279	GTTTTCTTCTGTTCTGTAACTGC	...*...*........*.*.	4	chr11
OT280	GTCTTCTCCTATTCTGCATCTGC	..**......*.......*.	4	chr7
OT281	GTCTTCTCCTATTCTGCATCTGC	..**......*.......*.	4	chr7
OT282	GTCTTCTCCTATTCTGCATCTGC	..**......*.......*.	4	chr7
OT283	GTCTTCTCCTATTCTGCATCTGC	..**......*.......*.	4	chr7
OT284	GTCCTCTCATCTTCTGCAGCTGA	..**....*.*.........	4	chr18
OT285	GCGATCTCCAGTTCTGTAGCTGC	.**......*......*...	4	chr17
OT286	GTTTGCTCCTGTCCTGCTGCTGC	...**.......*....*..	4	chr1
OT287	TTTACCTCCTCTTCTGCAGAGGT	*...*.....*........*	4	chr7
OT288	TTTATGTCCAGTTCTGAAGCTGT	*....*...*......*...	4	chr1
OT289	CTTAGCTCCAGTTCTGCTGCAGT	*...*....*.......*..	4	chr7
OT290	GCTCTCTCCTGATCAGCAGCTGC	.*.*.......*..*.....	4	chr13
OT291	GTTATCCTCTGGTCTGCACCTGC	......**...*......*.	4	chr18
OT292	TTTATCTTCTGTTCTGTACCTGT	*......*........*.*.	4	chr4
OT293	TTTATCTTCTCTTATGCAGCTGC	*......*..*..*......	4	chr5
OT294	GTTACCTTCCTTTCTGCAGCTGT	....*..*.**.........	4	chr1
OT295	CTTTTCTCCTGTGATGCAGCGGA	*..*........**......	4	chr17
OT296	CTTTTCTCATCTTCTGCAGCTGT	*..*....*.*.........	4	chr3
OT297	GGCATCTCTTGTTCTGCAGATGT	.**.....*..........*	4	chr9
OT298	TTTTTCTCCTGCTCTGGAGCAGA	*..*.......*....*...	4	chr2
OT299	GTTCTCTTCCGTTCTGCATCTGC	...*...*.*........*.	4	chr1
OT300	GTCATCACCTGATCTGCAGAGGT	..*...*....*.......*	4	chr14
OT301	GTTACCTGCTGTTCTGTAGATGT	....*..*........*..*	4	chr8
OT302	GTTATACCTTGTGCTGCAGCTGT	.....**.*...*.......	4	chr10
OT303	GTTATACCTTGTGCTGCAGCTGT	.....**.*...*.......	4	chr3

OT304	GGCATCTCTTGTTCTGCACCTGT	.**.....*.........*.	4	chr5
OT305	GTTTGCTCTTTTTCTGCAGCTGT	...**...*.*.........	4	chrX
OT306	GTTTTCTTCTGTTATGCAGAGGC	...*...*.....*.....*	4	chr11
OT307	GTCATTTCTTTTTCTGCAGCAGT	..*..*..*.*.........	4	chr1
OT308	GTTATCCTGTGTGCTGCAGCAGC	......***...*.......	4	chr1
OT309	GTTACCTGCTCTTATGCAGCTGC	....*..*..*..*......	4	chr10
OT310	GTTGTATCTTGTGCTGCAGCTGT	...*.*..*...*.......	4	chrX
OT311	GTTGTATCTTGTGCTGCAGCTGT	...*.*..*...*.......	4	chr9
OT312	GTTGTATCTTGTGCTGCAGCTGT	...*.*..*...*.......	4	chr7
OT313	CTTATCCCCTTGTCTGCAGCAGC	*.....*...**........	4	chr9
OT314	TTTCTCTCCTGGTTTGCAGCTGT	*..*.......*.*......	4	chr4
OT315	GTTGCCTCCGGTTCTGCAGTGGC	...**....*.........*	4	chr9
OT316	GTCATCCCTTGTTCTGCTGCTGT	..*...*.*........*..	4	chr15
OT317	TTAATCTCCTGGGCTGCAGCTGC	*.*........**.......	4	chr17
OT318	ATTCTCTCCAGTTCTGTAGCCGT	*..*.....*......*...	4	chr14
OT319	GTTAACTACTGTTTTGCTGCTGT	....*..*.....*...*..	4	chr1
OT320	GGTCTCTCCTGGTCAGCAGCAGC	.*.*.......*..*.....	4	chr15
OT321	ATTATCTTCTCTTATGCAGCTGT	*......*..*..*......	4	chr1
OT322	GTTGACTCCTGATCTGAAGCAGT	...**......*....*...	4	chr5
OT323	GTTTTCCCCTTTACTGCAGCAGT	...*..*...*.*.......	4	chr13
OT324	GTTCTGTCCTGTGCAGCAGCTGA	...*.*......*.*.....	4	chr7
OT325	GTTCTTTCCTCTTCAGCAGCAGC	...*.*....*...*.....	4	chr11
OT326	GTTCTCTGCTGGTCTGCAACGGC	...*...*...*......*.	4	chr7
OT327	GCTATCTACTGTTCTGCATTTGT	.*.....*..........**	4	chr9
OT328	GTAGTCTCCAGTGCTGCAGCTGT	..**.....*..*.......	4	chr7
OT329	GTTCCCTCCTCTTCTGAAGCTGC	...**.....*.....*...	4	chr7
OT330	GTCATTTCCTGTCCTGCAGTAGT	..*..*......*......*	4	chr9
OT331	GATATCACCTGTTCCGAAGCTGT	.*....*.......*.*...	4	chr14
OT332	GATCTCTCCTGTTCTGTACCTGC	.*.*............*.*.	4	chr6
OT333	CTTACCTCCTGTTCTGCACGTGA	*...*.............**	4	chr3
OT334	GTTAACTTCTGTGCTGCTGCTGC	....*..*....*....*..	4	chr16
OT335	TTCATCTCCTGTGCTGCTGCTGC	*.*.........*....*..	4	chr11
OT336	TTTCTCTCCTCTTCTGCTGCTGT	*..*......*......*..	4	chr11
OT337	GTTTCCTCCTCTTCCGCAGCTGT	...**.....*...*.....	4	chr6
OT338	GTGTTCTCCTGTTCTGATGCTGT	..**............**..	4	chr16
OT339	GCTCTCTCCTGTTCTGAAGAAGT	.*.*............*..*	4	chr3
OT340	GTTACCCCCTTTGCTGCAGCAGT	....*.*...*.*.......	4	chr14
OT341	GTGACCTCCTGTTCTGCCACTGT	..*.*............**.	4	chr4
OT342	GACATCTCCTGTTTTGCAGGTGA	.**..........*.....*	4	chr9
OT343	GATGTCTCCTGTTCTGCTGGAGA	.*.*.............*.*	4	chr6
OT344	ATTAACTCCTTTTATGCAGCAGT	*...*.....*..*......	4	chr3
OT345	CTTGTCTCCTGTTCTGGAGATGA	*..*............*..*	4	chr9
OT346	GTTTTCTGCTGTGCTGAAGCAGT	...*...*....*...*...	4	chr2
OT347	GTGCTCTCCTGTTTCGCAGCAGC	..**.........**.....	4	chr7
OT348	TTTGTCTCCTGTTCTGGAGTGGA	*..*............*..*	4	chr4
OT349	CTGATCTCCTGTGCTGCAGATGT	*.*.........*......*	4	chr7
OT350	GTGAGCTCCTGTTCTGGAGATGA	..*.*...........*..*	4	chr9
OT351	GTGATGTCCTGTTCTGCTGTTGT	..*..*...........*.*	4	chr6
OT352	GGTAACTCCTGTTCTGGAACAGC	.*..*...........*.*.	4	chr3
OT353	TTTATCTGCTGTTCAGGAGCAGT	*......*......*.*...	4	chr6
OT354	GTTTTATCCTGTTTGGCAGCTGT	...*.*.......**.....	4	chr10
OT355	GTTTTCTTCTGTTCAGCAGGGGT	...*...*......*....*	4	chr12

OT356	GTCCTCTCCTGTTCTGCCGATGT	..**.............*.*	4	chr4
OT357	GTCAGCTCCTGTCCTGGAGCTGT	..*.*.......*...*...	4	chr5
OT358	GTTTTGTCCTGTTCTGCATGTGT	...*.*............**	4	chrX
OT359	GTCATTTCCTGTGCTGCAGGTGC	..*..*......*......*	4	chr11
OT360	CTTCTCTCCTGTTAGGCAGCAGC	*..*.........**.....	4	chr4
OT361	GTACTCTCCTGTTCTCCAGGAGC	..**...........*...*	4	chrX
OT362	GTCTTCTCCTGTTCTCCAACTGA	..**...........*..*.	4	chr5
OT363	CTTTTCTCCTGTTCTTCAGAAGG	*..*...........*...*	4	chr10
OT364	TTTCTCTCCTTTTCTCCAGCTGT	*..*......*....*....	4	chr7
OT365	GGGATCTCCTGCTCTCCAGCAGA	.**........*...*....	4	chr9
OT366	GCTCTCTCCTGTTCCTCAGCAGA	.*.*..........**....	4	chr18
OT367	TTTATTTCCAGTTCTTCAGCTGG	*....*...*.....*....	4	chr1
OT368	GGTCTCTCCTGTAGTGCAGCTGC	.*.*........**......	4	chr3
OT369	GTCATCTTCTGTTGTGCAGATGT	..*....*.....*.....*	4	chr7
OT370	ATGATCTCCTCTTGTGCAGCAGG	*.*.......*..*......	4	chr17
OT371	GTTCTCCCCTGTTCTCCAGTTGT	...*..*........*...*	4	chr14
OT372	GTGTTCTCTTGTTCTTCAGCAGG	..**....*......*....	4	chr5
OT373	GTGACCTCTTGTTCTCCAGCTGT	..*.*...*......*....	4	chr10
OT374	GTTAGCACCTGTTGTGCAGGAGG	....*.*......*.....*	4	chr13
OT375	GTATTCTCCTGTTGGGCAGCTGA	..**.........**.....	4	chr2
OT376	GTCGTCTCCTATTCTCCAGCAGA	..**......*....*....	4	chr15
OT377	GTTATGTACTGCTGTGCAGCAGG	.....*.*...*.*......	4	chr5
OT378	GTTCCCTCCTGTTCTCCAGGGGC	...**..........*...*	4	chr19
OT379	GTATTCTCCTGTTGGGCAGCTGA	..**.........**.....	4	chr11
OT380	ATTGTCTCCTTTTCTTCAGCAGC	*..*......*....*....	4	chr9
OT381	GTTATCCTCTGTTCTTCAGATGC	......**.......*...*	4	chr1
OT382	CTCATCTCCTGCTCTTCAGCAGA	*.*........*...*....	4	chr17
OT383	GTAACCTCCTGTGCTTCAGCAGT	..*.*.......*..*....	4	chr3
OT384	ATTGTCTCCTGTTCCTCAGCCGC	*..*..........**....	4	chr12
OT385	GTTATTTTCTGTGCTTCAGCTGC	.....*.*....*..*....	4	chr15
OT386	GTTAACTGCTGTTGTGCAACTGT	....*..*.....*....*.	4	chr1
OT387	GGCATCTCCTGTTCTCCACCAGA	.**............*..*.	4	chr15
OT388	GTCCTCTCCAGTTCTCCAGCAGA	..**.....*.....*....	4	chr2
OT389	GTTTTCTGCTGTTGTGCTGCTGG	...*...*.....*...*..	4	chr1
OT390	GTTTTCTTCTGTTGTGGAGCTGT	...*...*.....*..*...	4	chr12
OT391	GTTGTGTCCTGTTGGGCAGCAGC	...*.*.......**.....	4	chr12
OT392	GTTTTCACCTGTTCTTCAGGAGA	...*..*........*...*	4	chr2
OT393	GATACCTCCTGTTCTTCATCTGT	.*..*..........*..*.	4	chr13
OT394	CTTGTCTCCTGTTCTTCTGCAGA	*..*...........*.*..	4	chr11
OT395	TTTATCTCCTTTTCTACAGCTGG	*.........*....*....	3	chr17
OT396	GTTGTCTCCAGTTCTGCATCTGG	...*.....*........*.	3	chr7
OT397	CTTATCTCCAGTTCTGTAGCAGG	*........*......*...	3	chr3
OT398	GTTATTTCCTATTCTGCAGTAGG	.....*....*........*	3	chr10
OT399	CTTATCTCCTGTTTTGCACCTGG	*............*....*.	3	chr18
OT400	CTTATCTCCTGTTTTGCACCTGG	*............*....*.	3	chr6
OT401	GTTATATCTTGTGCTGCAGCTGG	.....*..*...*.......	3	chr8
OT402	GTTATTTCCTGTTATGTAGCAGG	.....*.......*..*...	3	chr19
OT403	CTTATCTCCAGTTCTGCAACAGA	*........*........*.	3	chr19
OT404	GTTTTCTCCTGATCTGCAGGCGG	...*.......*.......*	3	chr4
OT405	TTTATCTCCAGTTCTGTAGCAGA	*........*......*...	3	chr16
OT406	GTTATATCCTGTACAGCAGCAGA	.....*......*.*.....	3	chr8
OT407	CTTATCTCCAGGTCTGCAGCAGA	*........*.*........	3	chr4

OT408	GTTATATCCTCTTCTGCTGCAGA	.....*....*......*..	3	chr1
OT409	GGTATCTCCTGTTTTGCAGTCGA	.*...........*.....*	3	chr9
OT410	GTTATCCCCAGCTCTGCAGCAGC	......*..*.*........	3	chr7
OT411	GTTATCACGTGCTCTGCAGCTGT	......*.*..*........	3	chr14
OT412	GTTATATCTTGTGCTGCAGCTGT	.....*..*...*.......	3	chr13
OT413	GTTAGCTCCTGTTCTGATGCCGC	....*...........**..	3	chr13
OT414	GTTGTCTCCTGGTCTGAAGCTGC	...*.......*....*...	3	chr5
OT415	GTTTTCTCCTCTTCTGCATCTGT	...*......*.......*.	3	chr14
OT416	GTTTTCTCCTTTTCTGCACCTGC	...*......*.......*.	3	chr2
OT417	GTTTTCTCATGTTCTGCAGGAGA	...*....*..........*	3	chr1
OT418	GTTATTTCCTCTGCTGCAGCTGT	.....*....*.*.......	3	chr6
OT419	GTTATCTGCTGTTTTGCAGGTGC	.......*.....*.....*	3	chr7
OT420	GTTGTCTCCTGTTCTCCATCAGG	...*...........*..*.	3	chr7
OT421	GTTTTCTCCTGCTGTGCAGCCGA	...*.......*.*......	3	chr11
OT422	GTTCTCTCCTGTTTTCCAGCAGG	...*.........*.*....	3	chr4
OT423	GTTATATCCTGTTTTTCAGCTGT	.....*.......*.*....	3	chr5
OT424	GTTATCTTCTGTTCTTCAGGAGT	.......*.......*...*	3	chr18
OT425	CTTATCTCCTGTTCTTCAGGTGT	*..............*...*	3	chr2
OT426	TTTATCTCTTGCTCTACAGCAGG	*.......*..*...*....	4	chr7
OT427	GTTATATCCTATACTGCTGCAGG	.....*....*.*....*..	4	chr1
OT428	GTTATCTTCAGTTTTGCTGCTGG	.......*.*...*...*..	4	chr16
OT429	TTTATCTCTTCTACTGCAGCAGG	*.......*.*.*.......	4	chr9
OT430	GTTTTCTCTTGCTCTGTAGCTGG	...*....*..*....*...	4	chr2
OT431	GATATCTCCTGTCCTGCTACAGG	.*..........*....**.	4	chr6
OT432	GTTATATCTTATGCTGCAGCTGG	.....*..*.*.*.......	4	chr8
OT433	GTTGTCTCTTGTCCTGTAGCAGG	...*....*...*...*...	4	chr15
OT434	GTTATCTTCTGTTCAGAAACAGG	.......*......*.*.*.	4	chr3
OT435	GTTATGTCATCATCTGCAGCAGG	.....*..*.**........	4	chr9
OT436	TTTATCTCCTGTACTGTACCTGG	*...........*...*.*.	4	chr3
OT437	GTTAACTCTATTTCTGCAGCAGG	....*...***.........	4	chr9
OT438	GTTCTCTCCAATTCTGAAGCAGG	...*.....**.....*...	4	chr2
OT439	GTTGTCTCTTGCGCTGCAGCTGG	...*....*..**.......	4	chr7
OT440	GGTATCTCCTGGTCAGTAGCTGG	.*.........*..*.*...	4	chr13
OT441	GTTATCCCCAGTGTTGCAGCTGG	......*..*..**......	4	chr3
OT442	GCTATCTCGTGTTCTGAACCAGG	.*......*.......*.*.	4	chr4
OT443	GTTAGCTCCTATGCTGCTGCTGG	....*.....*.*....*..	4	chr4
OT444	GTTCTCTCTTGTTCTGCTGATGG	...*....*........*.*	4	chr2
OT445	GTTCTCTCAAGTGCTGCAGCTGG	...*....**..*.......	4	chr2
OT446	GGTATCTCCAGTGCTGCAGTAGG	.*.......*..*......*	4	chr2
OT447	GTTGTCTCCAGTGCTGCGGCTGG	...*.....*..*....*..	4	chr2
OT448	GCTATCTCCTCTTCCGCAGAAGG	.*........*...*....*	4	chr9
OT449	GTTATTTCCTGTTTTACAGGAGG	.....*.......*.*...*	4	chr9
OT450	ATTATCTCTTGATCTGCATCTGA	*.......*..*......*.	4	chr4
OT451	CTTATCTCCAGCTCTGCAACAGA	*........*.*......*.	4	chr2
OT452	GTTATCTGTTGTTTTACAGCAGA	.......**....*.*....	4	chr10
OT453	GCTATCTCTTGTTTGGCAGCAGG	.*......*....**.....	4	chr11
OT454	GTTATCTTCTGTCTTGCATCTGA	.......*....**....*.	4	chr5
OT455	TTTATCTCATGCTCTGCGGCTGA	*.......*..*.....*..	4	chr6
OT456	GTTATCTGCTGATCAACAGCAGA	.......*...*..**....	4	chr13
OT457	GTTATGTCTTGATCTGCAGGGGG	.....*..*..*.......*	4	chr8
OT458	GTTTTCTCCCATTCTGGAGCTGG	...*.....**.....*...	4	chr16
OT459	CTTATCTCCAGTTCTATAGCAGA	*........*.....**...	4	chr1

OT460	GATATCTCTTGTTCTGGACCAGG	.*......*.......*.*.	4	chr4
OT461	CTTATCTCCAGCTCTGTAGCAGA	*........*.*....*...	4	chr10
OT462	CTTATCTCCAGCTCTGTAGCAGA	*........*.*....*...	4	chr4
OT463	GTTTTCTCCTGCTCTGGAGATGG	...*.......*....*..*	4	chr3
OT464	GTTATCACCTCTCCTGGAGCTGG	......*...*.*...*...	4	chr6
OT465	CTTATCTCTAGTTCTGTAGCAGA	*.......**......*...	4	chr19
OT466	GTTGTCTCTTGCTCTGTAGCTGA	...*....*..*....*...	4	chr9
OT467	GTGATCTCCTACTCTGCAGATGA	..*.......**.......*	4	chr4
OT468	GTTGTCTCTAGTTCTGCTGCTGA	...*....**.......*..	4	chr4
OT469	GTTATTTCCTGTAATACAGCTGA	.....*......**.*....	4	chr11
OT470	GTTATCTACTGTCCTGGTGCTGG	.......*....*...**..	4	chr14
OT471	GTTAACTCCAGCTCTGCAGAGGA	....*....*.*.......*	4	chr11
OT472	GTTATCACCTATGCTGGAGCAGG	......*...*.*...*...	4	chr1
OT473	GATATCTCCTATTTTGCAGTCGA	.*........*..*.....*	4	chr9
OT474	GATATCTCCTATTTTGCAGTCGA	.*........*..*.....*	4	chr9
OT475	CTTATCTCTTCTACTGCAGCAGA	*.......*.*.*.......	4	chr2
OT476	GTTGTCTCCTGATTTGCTGCAGA	...*.......*.*...*..	4	chr6
OT477	GTTTTCTCAGGTTCTGCATCTGA	...*....**........*.	4	chr4
OT478	CTTATCTCTTGCCCTGCAGCTGC	*.......*..**.......	4	chr14
OT479	GCTATCTCTTGCTCAGCAGCAGC	.*......*..*..*.....	4	chr1
OT480	GTTAGCTCTTGTTCTGTAGAGGA	....*...*.......*..*	4	chr6
OT481	CTTATCTCTTCTTCTGTAGCTGA	*.......*.*.....*...	4	chr3
OT482	CTTATCTCAGTTTCTGCAGCCGA	*.......***.........	4	chr7
OT483	GTAATCTCCATTTCTGTAGCAGA	..*......**.....*...	4	chr1
OT484	GTTAACTCATGTTCTGCAATTGA	....*...*.........**	4	chr7
OT485	GTTATGTCCGGTTTTGCAGAGGA	.....*...*...*.....*	4	chr10
OT486	GTTATGTCATGAGCTGCAGCTGA	.....*..*..**.......	4	chr6
OT487	GTTAGCTCCTTTTCTGCTACTGA	....*.....*......**.	4	chr9
OT488	GTTCTCTCGTGTACAGCAGCTGA	...*....*...*.*.....	4	chr13
OT489	CTTATCTCCAGCTCTGTAGCAGC	*........*.*....*...	4	chrX
OT490	GTTTTCTCTTGCTCTGCAGAGGC	...*....*..*.......*	4	chr15
OT491	GTCATCTCAGGTTCTGCACCTGA	..*.....**........*.	4	chr3
OT492	GTTTTCTCCTGTTCTGGCTCTGG	...*............***.	4	chr19
OT493	GTTCTCTCAAGTGCTGCAGCAGA	...*....**..*.......	4	chr1
OT494	GTTCTCTCAAGTGCTGCAGCAGA	...*....**..*.......	4	chr7
OT495	GTTGTCTCTTTTTCTGCAGTTGA	...*....*.*........*	4	chr6
OT496	GTTATATCCCATTCTGTAGCTGT	.....*...**.....*...	4	chr5
OT497	GTTATCTTCTGAACTGTAGCAGT	.......*...**...*...	4	chr19
OT498	GTTATCTGCTGCTCTGCCCCTGA	.......*...*.....**.	4	chr4
OT499	GTTCTCTCCTTTTATACAGCAGA	...*......*..*.*....	4	chr3
OT500	GTTGTCTCCTTTGTTGCAGCTGA	...*......*.**......	4	chr9
OT501	TTTATCTCCTCTTCTGAATCAGC	*.........*.....*.*.	4	chr9
OT502	GTTTTCTCCTGTTCTATACCTGC	...*...........**.*.	4	chr14
OT503	GTTATCTGCCTTTCTGCAGTCGA	.......*.**........*	4	chr10
OT504	GTTATCTGCTATTATGCAACAGT	.......*..*..*....*.	4	chr4
OT505	GTTATTTCCATTTCTGAAGCTGT	.....*...**.....*...	4	chr11
OT506	GTTGTCTCTTATGCTGCAGCCGT	...*....*.*.*.......	4	chr1
OT507	GTTAGCTCCCCCTCTGCAGCTGT	....*....***........	4	chr19
OT508	GTTGTCTCGTGTTCTGCAAATGT	...*....*.........**	4	chr14
OT509	TTTATCTCCTCTTCTACAGTGGT	*.........*....*...*	4	chr4
OT510	GTTTTCTCTAGTTCTGGAGCTGA	...*....**......*...	4	chr4
OT511	GTTAGCTCCTGTCCTGTAGAAGT	....*.......*...*..*	4	chr19

OT512	GTTACCTCCTGCTCTGTCGCTGC	....*......*....**..	4	chr5
OT513	GTTAACTCTTTTTCTGCTGCAGC	....*...*.*......*..	4	chr13
OT514	GTTCTCTCCTGCTCTGCAATAGC	...*.......*......**	4	chr2
OT515	GTTATGTCCTGTTCAGAAACAGT	.....*........*.*.*.	4	chr7
OT516	GTTAACTCCTTTTCTGCTTCAGC	....*.....*......**.	4	chr8
OT517	GTCATCTCCTGTCCTGCTGAGGT	..*.........*....*.*	4	chr4
OT518	GTGATCTCTTTTTCTGCTGCAGT	..*.....*.*......*..	4	chr7
OT519	GTTATTTCCTTTTCTGTTGCTGT	.....*....*.....**..	4	chr4
OT520	GTTCTCTCCTGTGCTGCATTTGA	...*........*.....**	4	chr3
OT521	GTTATATCCTCTGTTGCAGCAGT	.....*....*.**......	4	chr10
OT522	GTCATCTCCTCTTCAGAAGCAGC	..*.......*...*.*...	4	chrX
OT523	GTTCTCTCCTCCTCTGCACCAGT	...*......**......*.	4	chr4
OT524	GTTATCTTCTGTTCCAGAGCTGA	.......*......***...	4	chr13
OT525	GTGATCTCCTGTTCTACCCCTGC	..*............*.**.	4	chr8
OT526	ATTATCTCCTTTTATGTAGCTGT	*.........*..*..*...	4	chr16
OT527	GTTATGTCTTGTTCTGGAGAAGA	.....*..*.......*..*	4	chr14
OT528	GTTTTCTCCTGTGCTGCTTCTGT	...*........*....**.	4	chr18
OT529	GTTATATCCTATCCTGCAGGAGT	.....*....*.*......*	4	chr2
OT530	GTTTTCTCCGTTTCTGCAGTTGT	...*.....**........*	4	chr14
OT531	GTTTTCTCCTTTGCTGCAGTGGC	...*......*.*......*	4	chr14
OT532	GTTGTCTCCTGGCCTGCAGGAGC	...*.......**......*	4	chr3
OT533	GTTCTCTCCTCTGCTGCTGCAGT	...*......*.*....*..	4	chr2
OT534	CTTATCTCCTATTCTGAAGGGGT	*.........*.....*..*	4	chrX
OT535	GTTATATCCTTTTCTGCATGAGC	.....*....*.......**	4	chr5
OT536	GTTAACTCCAGTTCAGGAGCTGT	....*....*....*.*...	4	chr19
OT537	GTTCTCTCCTGCTTGGCAGCTGT	...*.......*.**.....	4	chr13
OT538	GTCATCTCTTGTTCTGCTGGGGT	..*.....*........*.*	4	chr13
OT539	GTGATCTCCTGTTCTGGACTAGT	..*.............*.**	4	chr6
OT540	TTTATCTCCTGATCTTTAGCAGA	*..........*...**...	4	chrX
OT541	GTTTTCTCCAGTTCTTCACCTGA	...*.....*.....*..*.	4	chr15
OT542	GTTATTTCCTGTTCTCCACTCGC	.....*.........*..**	4	chrX
OT543	GATATCTCCTGTTCTCCGGGAGA	.*.............*.*.*	4	chr7
OT544	TTTATCTCCTGTTCTTCTGTTGA	*..............*.*.*	4	chr4
OT545	GTTACCTCCTGTTCATCACCTGG	....*.........**..*.	4	chr7
OT546	TTTATCTCCTGTCCTCCATCCGT	*...........*..*..*.	4	chr10
OT547	GTTTTCTCATGTTGAGCAGCTGT	...*....*....**.....	4	chr2
OT548	GTTCTCTCCTTGTCTCCAGCAGC	...*......**...*....	4	chr2
OT549	GTTACCTCCTGTTCTCCTTCAGG	....*..........*.**.	4	chr14
OT550	TTTATCTCCTGTTGTGGATCAGA	*............*..*.*.	4	chr9
OT551	GATATCTCCTGTTGTGGATCCGG	.*...........*..*.*.	4	chr13
OT552	CTTATCTCCTGTGGTGCTGCTGT	*...........**...*..	4	chr2
OT553	GTTTTCTCATGTTTTCCAGCTGC	...*....*....*.*....	4	chr1
OT554	GTTATATCTTGTACTTCAGCAGA	.....*..*...*..*....	4	chr6
OT555	GTTATCTAGTGTTCTTCAGATGC	.......**......*...*	4	chr15
OT556	GTTGTCTCCTCATCTTCAGCAGC	...*......**...*....	4	chr9
OT557	GCTATCTCCTGTTGTTGAGCTGG	.*...........*.**...	4	chr2
OT558	GTTATCTACTGTTTATCAGCAGT	.......*.....***....	4	chr9
OT559	GTTGTCTCCTTTTGAGCAGCTGT	...*......*..**.....	4	chr10
OT560	GTTATCTTCTGGTGTGCAGATGT	.......*...*.*.....*	4	chr16
OT561	GTCATCTCCTGTGCTCCATCAGT	..*.........*..*..*.	4	chr2
OT562	GTTTTCTCCTTTTCACCAGCTGG	...*......*...**....	4	chr3
OT563	GTTCTCTCCTGTACTCCAGATGA	...*........*..*...*	4	chr13

OT564	GATATCTCCCTTTCTCCAGCAGC	.*.......**....*....	4	chrX
OT565	TTTATCTCCATTTCTCCAGCTGG	*........**....*....	4	chr8
OT566	GTTCTCTCCTGTTGTCAAGCAGG	...*.........*.**...	4	chrX
OT567	CTTATCTCCTACTGTGCAGCAGG	*.........**.*......	4	chr16
OT568	GTTATCTTCTGTGATCCAGCAGT	.......*....**.*....	4	chr4
OT569	GTTATCTTGTGTTCCTCAGCTGG	.......**.....**....	4	chr4
OT570	GTTATATCCTGTTCCTCAGGTGA	.....*........**...*	4	chrY
OT571	GTTATCTTCTGATCTCCACCAGT	.......*...*...*..*.	4	chr5
OT572	GTTCTCTCCTGTTCCTCTGCAGG	...*..........**.*..	4	chr9
OT573	TTTATCTCCTTTTCTTCAACTGA	*.........*....*..*.	4	chr4
OT574	GTTCTCTCCTGTTGTGGAACAGG	...*.........*..*.*.	4	chr2
OT575	GTTATCTTCTGTTCTTCACACGA	.......*.......*..**	4	chr6
OT576	GCTATCTCCAGTTCTTCATCTGC	.*.......*.....*..*.	4	chr2
OT577	GTTATGTCCTATTCTTCACCTGT	.....*....*....*..*.	4	chr19
OT578	GTTACCTCCCTTTCTTCAGCAGT	....*....**....*....	4	chr14
OT579	GTTAGCTCCAGTTCCTCAGCAGG	....*....*....**....	4	chr6
OT580	TTTATCTCTTGTTGAGCAGCAGC	*.......*....**.....	4	chr4
OT581	GTCATCTCTTTTTGTGCAGCAGA	..*.....*.*..*......	4	chr3
OT582	GTTATGTCCTGCTCTTGAGCTGC	.....*.....*...**...	4	chr9
OT583	GTGATCTCCTGTACTCCAGAAGT	..*.........*..*...*	4	chr7
OT584	GTTATCTCCTCTTCTGCTGAGGG	..........*......*.*	3	chr13
OT585	GTTATCTCATGTCCTGTAGCTGA	........*...*...*...	3	chr9
OT586	GTTATCTCCTGTTCTTTAACAGT	...............**.*.	3	chr4
OT587	GTTATCTCCTGTTGTGGATCCGG	.............*..*.*.	3	chr13
OT588	GTTATCTCCAGCTCTCCAGCAGA	.........*.*...*....	3	chr6
OT589	GTTATCTCATGTGCTTCAGCAGT	........*...*..*....	3	chr1
OT590	GTTATCTCCAGAACTGAAGCAGG	.........*.**...*...	4	chr16
OT591	GTTATCTCCTGCTTCGAAGCTGG	...........*.**.*...	4	chr9
OT592	GTTATCTCAAGTTCTATAGCTGA	........**.....**...	4	chr4
OT593	GTTATCTCCTGGTTTGGAACTGG	...........*.*..*.*.	4	chr8
OT594	GTTATCTCCAGTTCTGAATAGGA	.........*......*.**	4	chr7
OT595	GTTATCTCCTCTCCTGCAAACGA	..........*.*.....**	4	chr6
OT596	GTTATCTCCTGATCTGCCAGGGG	...........*.....***	4	chr7
OT597	GTTATCTCTTGGCCTGCAGAAGT	........*..**......*	4	chr19
OT598	GTTATCTCCAGATCTGCCACTGT	.........*.*.....**.	4	chr13
OT599	GTTATCTCACCTTCTGTAGCAGT	........***.....*...	4	chr16
OT600	GTTATCTCCTGGTTTGAACCAGT	...........*.*..*.*.	4	chr6
OT601	GTTATCTCCATTTATGAAGCAGC	.........**..*..*...	4	chr8
OT602	GTTATCTCCATTTATGAAGCAGC	.........**..*..*...	4	chr8
OT603	GTTATCTCCTGTGTTGCTGAGGT	............**...*.*	4	chr17
OT604	GTTATCTCCTGAGTTGCAGGGGA	...........***.....*	4	chrX
OT605	GTTATCTCCTGCTCTAGAGAAGC	...........*...**..*	4	chr2
OT606	GTTATCTCATCTTATGCAGGAGA	........*.*..*.....*	4	chr1
OT607	GTTATCTCCTGTTACACAGTGGT	.............***...*	4	chr18
OT608	GTTATCTCAGGTACTGGAGCTGC	........**..*...*...	4	chr5
OT609	GTTATCTCCTCATTTGCAGGTGC	..........**.*.....*	4	chr3
OT610	GTTATCTCCTTTTCTGGCCCTGA	..........*.....***.	4	chrX
OT611	GTTATCTCCTTTCCCGCAGGTGC	..........*.*.*....*	4	chr11
OT612	GTTATCTCCTGTTCTTGGTCAGG	...............****.	4	chr19
OT613	GTTATCTCCTGTGGGGCAGTGGT	............***....*	4	chr19
OT614	GTTATCTCCTATTGTGCGACAGA	..........*..*...**.	4	chr9
OT615	GTTATCTCCTAGTCTTCGGCTGG	..........**...*.*..	4	chr4

OT616	GTTATCTCCTTTTATTCAGTGGT	..........*..*.*...*	4	chr11
OT617	GTTATCTCATGCTGTGCATCAGC	........*..*.*....*.	4	chr17
OT618	GTTATCTCTTGTTCTCTTGCTGA	........*......***..	4	chr1
OT619	GTTATCTCCTGTTTTTTATCTGA	.............*.**.*.	4	chr9
OT620	GTTATCTCCTGTTCCCATGCAGA	..............****..	4	chr7
OT621	GTTATCTCCTGTTGTGGATTGGA	.............*..*.**	4	chr2

Supplementary Table S4. List of 22 off-target RNA editing loci in the heart of *mdx*^{4cv} mice received AAV9-iNG.

Chromosome	Position	REF	ALT	Genomic Context	Gene
**chr19**	4004576	A	G	GAGCT**A**CGAGT	Doc2g
**chr8**	15069397	A	G	TTCTT**A**CGGAG	Myom2
**chr16**	29653621	A	G	TGTGT**A**CGAAA	Opa1
**chr13**	11721772	T	C	GCTCT**A**CGAGA	RyR2
**chr16**	20687388	A	G	AAATT**A**CGAAG	Eif4g1
**chr2**	132309669	A	G	CATGC**A**TTATC	Cds2
**chr14**	54979236	T	C	GAGAA**A**AGCGA	Myh7
**chr4**	156219748	A	G	GAACT**A**CGGAG	Perm1
**chr1**	183357354	T	C	GCCAT**A**CGAAG	Mia3
**chr4**	151980760	A	G	AGGCT**A**CGAAA	Dnajc11
**chr4**	128752175	A	G	CCTGT**A**CCCTC	Phc2
**chr9**	65346783	A	G	CCACT**A**CGAGC	Pdcd7
**chr2**	90917460	T	C	GGAGT**A**CGAGA	Ptpmt1
**chr8**	47864523	T	C	GGTCT**A**CGAAG	Wwc2
**chr4**	115738609	A	G	TGGCT**A**CGAAC	Efcab14
**chr14**	55629144	T	C	AGCTT**A**CGAAG	Ipo4
**chr1**	119910367	T	C	GCTCT**A**CGGAG	Tmem177
**chr4**	53561051	A	G	CATTT**A**CGAAA	Slc44a1
**chr2**	25446990	A	G	ACCAT**A**CGAAG	Abca2
**chr7**	44505379	T	C	TGTCT**A**CGAAC	Mybpc2
**chr11**	65002693	T	C	ACCCT**A**CGTAG	Arhgap44
**chr5**	135736272	T	C	GCCAT**A**CGATG	Tmem120a

Supplementary Table S5. List of gRNA target sequences and primers for PCR in this study.

Name	Sequence
**Mdx4cv-gRNA**	GTTATCTCCTGTTCTGCAGC
**NT-gRNA**	GTTTATGTCACCAGAGTAAC
**mDMD-i52-F**	GAGGTAATAGAGCCAAGCCCT
**mDMD-i53-R**	GCAAGAATTCCACTTTTCACTTCCT
**mDMD-E51-F**	CTGTCATCTCCAAACTAGAAATGC
**mDMD-E55-R**	GCAGCCTCTTGCTCACTTACTC
**S1-gRNA**	GATGACAGGCAGGGGCACCG
**S1-F**	TTCCAGTGGTTCAATGGTCA
**S1-R**	CTTTCAACCCGAACGGAGAC
**S2-F**	TCCTGTCCAAATGCAGCTTA
**S2-R**	TCCTGCAGTCTATGCCTCTC
**VEGFA-S5-gRNA**	GAGCGAGCAGCGTCTTCGAG
**VEGFA-S12-gRNA**	GCAGACGGCAGTCACTAGGG
**VEGFA-S14-gRNA**	GGGAAGCTGGGTGAATGGAG
**VEGFA-F**	AGCTGTTTGGGAGGTCAGAA
**VEGFA-R**	AGGGAGCAGGAAAGTGAGGT
**Site13-gRNA**	GTCGCAGGACAGCTTTTCCT
**Site13-F**	TGTAGCTACGCCTGTGATGG
**Site13-R**	TGCCCTGAGATCTTTTCCTC
**FANCF-gRNA**	GATCCAGGTGCTGCAGAAGG
**FANCF-F**	CTCTTGCCTCCACTGGTTGT
**FANCF-R**	TCGGTAGGATGCCCTACATC
**Q623X-gRNA**	ATCCTACAGCATGGTGGCTG
**Puro-F**	AGTGGTCTCCGGAAACCTCCGCGCCCCGCAAC
**GFP-R**	TCCTTGAAGAAGATGGTGCG

Supplementary Table S6. List of plasmids used in this study.

ID	Name	Description
**pXL-0570**	pCMV_ABEmax (Addgene # 112095)	Expressing ABEmax; used in Fig. 1c-e; Fig. 4b,d; Supplementary Fig. S2
**pXL-0550**	pCMV_xCas9_3.7_-ABE_7.10 (Addgene #108382)	Expressing ABE-x; used in Fig. 1c-e; Fig. 4b; Supplementary Fig. S2.
**pXL-0645**	pCMV_ABEmaxNG	Expressing ABE-NG; used in Fig. 1c-e; Fig. 2a-f; Fig. 3b; Fig. 4b-d;
**pXL-0752**	pLKO-puro-2A-mdx4cv-GFP	*mdx*^{4cv} reporter; used in Fig. 1c-e; Fig. 4c, d, h; Supplementary Fig. S1.
**pXL-0631**	pLenti-puro-OgRNA_mdxE53	gRNA targeting *mdx*^{4cv} mutation; used in Fig. 1c-e; Fig. 3b-d; Fig. 4c-h; Fig. 7g,h; Supplementary Fig. S1.
**pXL-0858**	pCMV_ABEmaxNG-NGA	Expressing ABE-NGA; used in Fig. 2a-f.
**pXL-0869**	pCMV_ABE-NGC	Expressing ABE-NGC; used in Fig. 2a-f.
**pXL-0872**	pCMV_ABE-NG-loop	Expression ABE-NG-loop; used in Fig. 2a-f.
**pXL-0712**	pCMV-ABEmaxNGX	Expressing ABE-NGX; used in Fig. 2a-f.
**pXL-0868**	pCMV-ABEmaxNGX-NGA	Expressing ABE-NGX-NGA; used in Fig. 2a-f.
**pXL-0875**	pCMV-ABEmaxNGX-NGC	Expressing ABE-NGX-NGC; used in Fig. 2a-f.
**pXL-0877**	pCMV-ABEmaxNGX-loop	Expressing ABE-NGX-loop; used in Fig. 2a-f.
**pXL-0723**	pCMV-ABEmaxSC	Expressing ABEmaxSc; used in Fig. 2a-f.
**pXL-0627**	pLenti-puro-S1OgRNA	S1 targeting gRNA; used in Fig. 2a.
**pZC0009**	pLenti-Q2440X-ogRNA	Non-targeting gRNA; used in Supplementary Fig. S3.
**pXL-0796**	pLenti-VEGFA-S5	gRNA targeting VEGFA Site5; used in Fig. 2b.
**pXL-0797**	pLenti-VEGFA-S12	gRNA targeting VEGFA Site12; used in Fig. 2c.
**pXL-0798**	pLenti-VEGFA-S14	gRNA targeting VEGFA Site14; used in Fig. 2d.
**pXL-0800**	pLenti-Site13	gRNA targeting Site13; used in Fig. 2e.
**pXL-0801**	pLenti-FANCF-ogRNA	gRNA targeting FANCF; used in Fig. 2f.
**pZC0103**	pCMV_miniABE_NG(V82G)	Expressing miniABE(82G)-NG; used in Fig. 3b,c.
**pZC0104**	pCMV_ miniABE-NG	Expressing miniABE-NG; used in Fig. 3b-d.
**pXL-0853**	pCMV_miniABEmax-NG(A56G)	Expressing miniABE(A56G)-NG; used in Fig. 3b,c.
**pXL-0854**	pCMV_miniABEmaxNG(GG)	Expressing miniABE(GG)-NG; used in Fig. 3b-d; Supplementary Fig. S1.
**pXL-0420**	pCMV_ABE7.10 (Addgene #102919)	Expressing ABE7.10; used in Fig. 4b; Supplementary Fig. S2.
**pXL-0670**	pBac-rAAV-ABEmaxN-E53 OgRNA	Expressing Cfa Split_N of ABE and mdx4cv gRNA; used in Fig. 4b-d.
**pXL-0671**	pBac-rAAV-ABEmaxC-NG-E53 OgRNA	Expressing Cfa Split_C of ABE-NG and mdx4cv gRNA; used in Fig. 4b-d.
**pXL-0672**	pBac-rAAV-ABEmaxN2-E53 OgRNA	Expressing Gp41-1 Split_N of ABE and mdx4cv gRNA; used in Fig. 4b-d.
**pXL-0673**	pBac-rAAV-ABEmaxC2-NG-E53 OgRNA	Expressing Gp41-1 Split_C of ABE-NG and mdx4cv gRNA; used in Fig. 4b-d.

**pZC0117**	pX601-mhCMV-ABEmaxNGA-C3-E53ogRNA	Expressing Npu Split_C of iABE-NGA and mdx4cv gRNA; used in Fig. 4e-h.
**pZC0118**	pX601-mhCMV-miniABEmax-N3-E53ogRNA	Expressing Npu Split_N of iABE-NGA and mdx4cv gRNA; used in Fig. 4e-h.
**pZC0119**	pX601-mhCMV-miniABEmax-N3-zeo	Expressing Npu Split_N of iABE-NGA; used in Fig. 4e-h.
**pZC0031**	pX601-mhCMV-miniABEmax-N2-E53ogRNA	Expressing Gp41-1 Split_N of iABE-NGA and mdx4cv gRNA; used in Fig. 4e-h.
**pZC0033**	pX601-mhCMV-ABEmaxNGA-C2-E53ogRNA	Expressing Gp41-1 Split_C of iABE-NGA and mdx4cv gRNA; used in Fig. 4e-h.
**pXL-0855**	pX601-MHP1-miniABEmaxNG-N2-(GG)	Expressing Gp41-1 Split_N of iABE-NGA and mdx4cv gRNA, MHP1 promoter; used for AAV9 production in Fig. 5-7.
**pXL-0706**	pX601-MHP1-ABEmaxC2-NG-E53 ogRNA	Expressing Gp41-1 Split_C of iABE-NGA and mdx4cv gRNA, MHP1 promoter; used for AAV9 production in Fig. 5-7.
**pYZ1059**	pCMV_NG-ABE8e	Expressing ABE8e-NG; used in Supplementary Fig. S1
**pZC0105**	pCMV_NG-ABE8.17	Expressing ABE8.17-NG; used in Supplementary Fig. S1
**pZC0106**	pCMV_NG-ABE8.20	Expressing ABE8.20-NG; used in Supplementary Fig. S1
**pZC0111**	pCMV_SpG-ABE8e(V106W)	Expressing ABE8e-SpG(V106W); used in Supplementary Fig. S1
**pPW-0007**	pLKO-puro-2A-Q623X-GFP	Q623X reporter; used in Fig. S1b.
**pPW-0010**	pLenti-Q623X-ogRNA	Q623X targeting gRNA; used in Fig. S1b.
**pXL-0419**	pCMV-ABE7.9 (Addgene #102918)	Expressing ABE7.9; used in Fig. S2.
**pXL-0419**	pCMV-ABE7.9 (Addgene #102918)	Expressing ABE7.9; used in Fig. S2.
**pYG9010**	pBac-rAAV-IntC-SpCas9n	Expressing Npu_N-SpCas9 nickase; used in Fig. S2.
**pYG9011**	pBac-rAAV-ABE7.10v3	Expressing GFP-TadA-TadA*-Npu_C; used in Fig. S2.

Supplementary Table S7. List of primers used for NGS in this study.

Name	Sequence
**Mdx4cv-E52-F**	ACACTCTTTCCCTACACGACGCTCTTCCGATCTGAACTCATTACTGCTGCCCAGA
**Mdx4cv-E53-R**	GTGACTGGAGTTCAGACGTGTGCTCTTCCGATCGACCTGTTCGGCTTCTTCCTTA
**Mdx4cv-i52-F**	ACACTCTTTCCCTACACGACGCTCTTCCGATCTAAATTTCCACTGTCTTCTCTTGAGT
**Mdx4cv-i53-R**	GTGACTGGAGTTCAGACGTGTGCTCTTCCGATCGCTTGCCTCTGACCTGTCCTAT
**mChr16OT-F**	ACACTCTTTCCCTACACGACGCTCTTCCGATCTGTGACTAGGGGCAAAGCAAGAT
**mChr16OT-R**	GTGACTGGAGTTCAGACGTGTGCTCTTCCGATCCTTCCAAACTTTCTGCCCATTC
**mChr1OT-F**	ACACTCTTTCCCTACACGACGCTCTTCCGATCTAACACAGCGTGCTCTTTCCTTAC
**mChr1OT-R**	GTGACTGGAGTTCAGACGTGTGCTCTTCCGATCGTTCAGAAGAACATCCCGTTGAC
**mChr13OT1-F**	ACACTCTTTCCCTACACGACGCTCTTCCGATCTATGTTTTCCAAAGGCCACAG
**mChr13OT1-R**	GTGACTGGAGTTCAGACGTGTGCTCTTCCGATCGAAAACAGATACAGGGCTGGT
**mChr5OT2-F**	ACACTCTTTCCCTACACGACGCTCTTCCGATCTTTCTAAGCCAGGCAGACACA
**mChr5OT2-R**	GTGACTGGAGTTCAGACGTGTGCTCTTCCGATCGTTTCTGGATGCACCAGGTT
**mChr3OT3-F**	ACACTCTTTCCCTACACGACGCTCTTCCGATCTAGGGTGGAAGCAGGAAAGAT
**mChr3OT3-R**	GTGACTGGAGTTCAGACGTGTGCTCTTCCGATCGGCAAACTTTTCAACCCTCA
**mChr8OT4-F**	ACACTCTTTCCCTACACGACGCTCTTCCGATCTCTCTGAAGAGGGCATCCAAC
**mChr8OT4-R**	GTGACTGGAGTTCAGACGTGTGCTCTTCCGATCTACATGTCCGTGCTGGTTGT
**mChr5OT5-F**	ACACTCTTTCCCTACACGACGCTCTTCCGATCTTGGTCAGCTGTCTCATGCTC
**mChr5OT5-R**	GTGACTGGAGTTCAGACGTGTGCTCTTCCGATCCCCATTGCTGTCACTTCTGA
**NGS-final-F**	AATGATACGGCGACCACCGAGATCTACACTCTTTCCCTACACGAC
**NGS-final-R1**	CAAGCAGAAGACGGCATACGAGATCTTGTAGTGACTGGAGTTCAGACGT
**NGS-final-R2**	CAAGCAGAAGACGGCATACGAGATCAGATCGTGACTGGAGTTCAGACGT
**NGS-final-R3**	CAAGCAGAAGACGGCATACGAGATCCGTCCGTGACTGGAGTTCAGACGT
**NGS-final-R4**	CAAGCAGAAGACGGCATACGAGATATGTCAGTGACTGGAGTTCAGACGT
**NGS-final-R5**	CAAGCAGAAGACGGCATACGAGAT GTCCGC GTGACTGGAGTTCAGACGT
**NGS-final-R6**	CAAGCAGAAGACGGCATACGAGAT TTAGGC GTGACTGGAGTTCAGACGT
**NGS-final-R7**	CAAGCAGAAGACGGCATACGAGAT CGATGT GTGACTGGAGTTCAGACGT
**NGS-final-R8**	CAAGCAGAAGACGGCATACGAGAT TGACCA GTGACTGGAGTTCAGACGT
**NGS-final-R9**	CAAGCAGAAGACGGCATACGAGAT AGTCAA GTGACTGGAGTTCAGACGT
**NGS-final-R10**	CAAGCAGAAGACGGCATACGAGAT AGTTCC GTGACTGGAGTTCAGACGT
**NGS-final-R11**	CAAGCAGAAGACGGCATACGAGAT GATCAG GTGACTGGAGTTCAGACGT
**NGS-final-R12**	CAAGCAGAAGACGGCATACGAGAT ACAGTG GTGACTGGAGTTCAGACGT
**NGS-final-R13**	CAAGCAGAAGACGGCATACGAGAT TATACT GTGACTGGAGTTCAGACGT
**NGS-final-R14**	CAAGCAGAAGACGGCATACGAGAT CAACAA GTGACTGGAGTTCAGACGT
**NGS-final-R15**	CAAGCAGAAGACGGCATACGAGAT GTTGTT GTGACTGGAGTTCAGACGT
**NGS-final-R16**	CAAGCAGAAGACGGCATACGAGAT TCGGTT GTGACTGGAGTTCAGACGT
**NGS-final-R17**	CAAGCAGAAGACGGCATACGAGAT AGTATT GTGACTGGAGTTCAGACGT
**NGS-final-R18**	CAAGCAGAAGACGGCATACGAGAT TCTTGT GTGACTGGAGTTCAGACGT

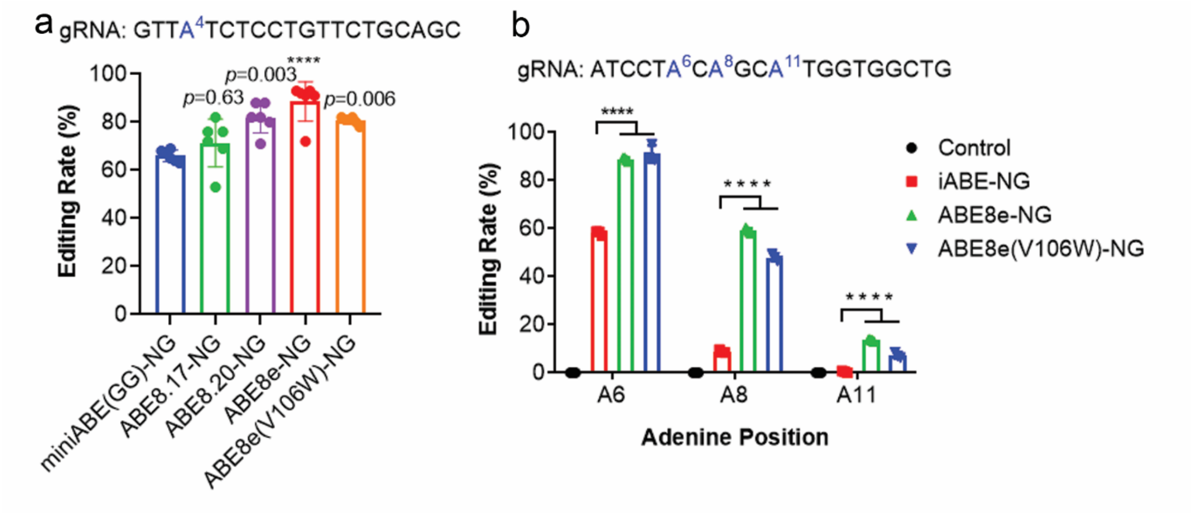

Supplementary Fig. S1. Comparison of the editing efficiency of different ABEs at the *mdx^{4cv}* target site (**a**) and human dysferlin Q605X site (**b**). All base editors carry the SpCas9-NG and differ at the adenine deaminase domain. n=6 wells/group for panel **a**; n=3 wells/group for panel **b**; **** $p < 0.0001$ (one-way ANOVA with Turkey's multiple comparisons test for panel **a**; two-way ANOVA with Turkey's multiple comparisons test for panel **b**). Data are mean ± SD.

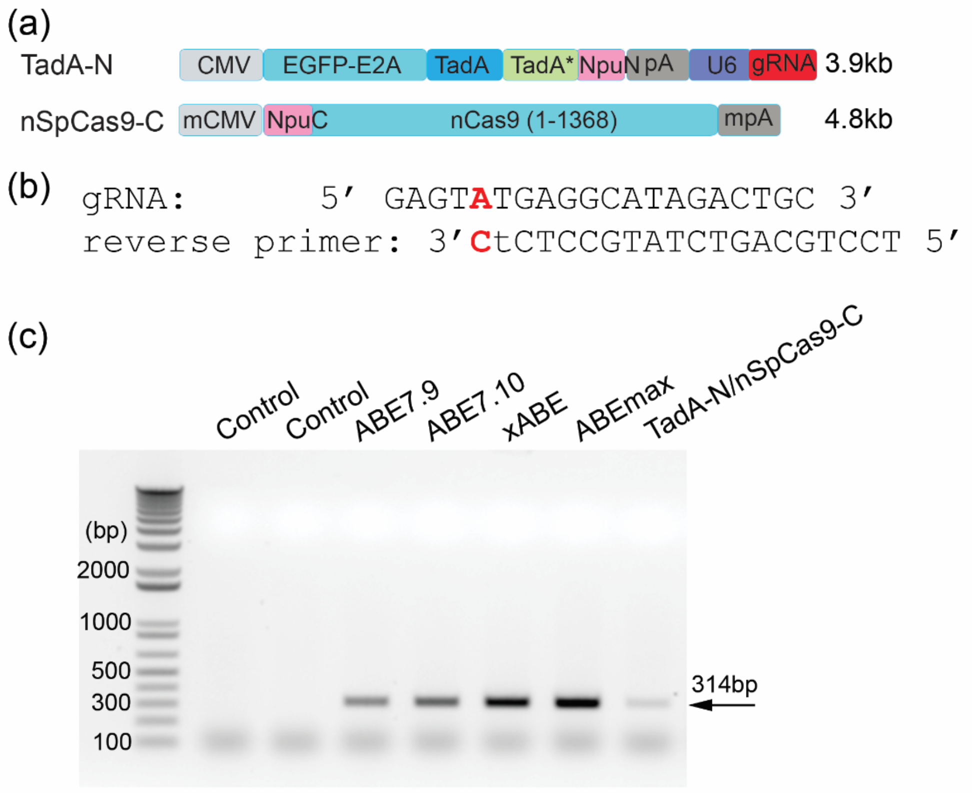

Supplementary Fig. S2. The intein split of ABEmax had relatively low editing activity. (a) Schematics showing the two halves of intein-split ABEmax. The TadA-TadA* was fused with Npu intein N-terminal fragment and SpCas9 nickase (nSpCas9) was fused with Npu intein C-terminal fragment. (b) The sequences of the S2-gRNA and the reverse primer designed to specifically amplify the mutated DNA. This reverse primer could not amplify the WT allele because it has two mismatches at the end. (c) Genomic DNA PCR genotyping analysis of HEK293 cells at 5 days after transfection with S2-gRNA and different versions of ABEs. The arrow indicates the PCR amplicon with the targeted point mutation. This experiment was repeated independently for 3 times.

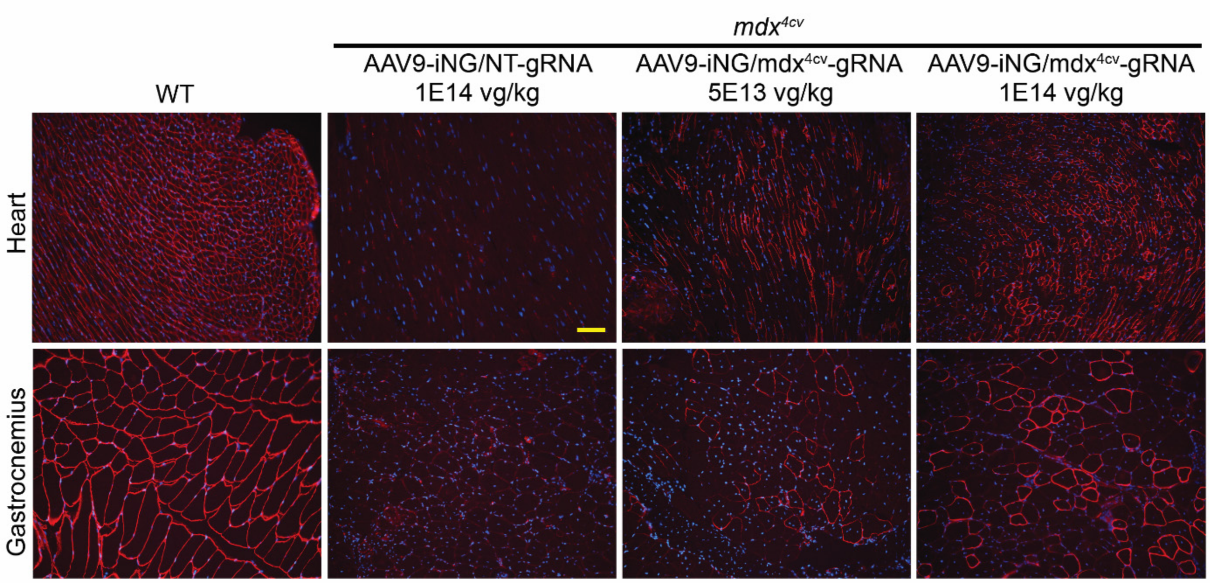

Supplementary Fig. S3. Immunofluorescence staining of heart and gastrocnemius muscle sections with anti-dystrophin antibody and DAPI. The *mdx*^{4cv} mice at 5 weeks of age received either 5x10^{13}, 1x10^{14} AAV9-iNG/mdx^{4cv}-gRNA or 1x10^{14} vg/kg AAV9-iNG/non-targeting-gRNA (NT-gRNA) through tail vein injection, and sacrificed at 10 weeks of age for immunofluorescence staining analysis. Scale bar: 100 µm. n=3 mice/group.

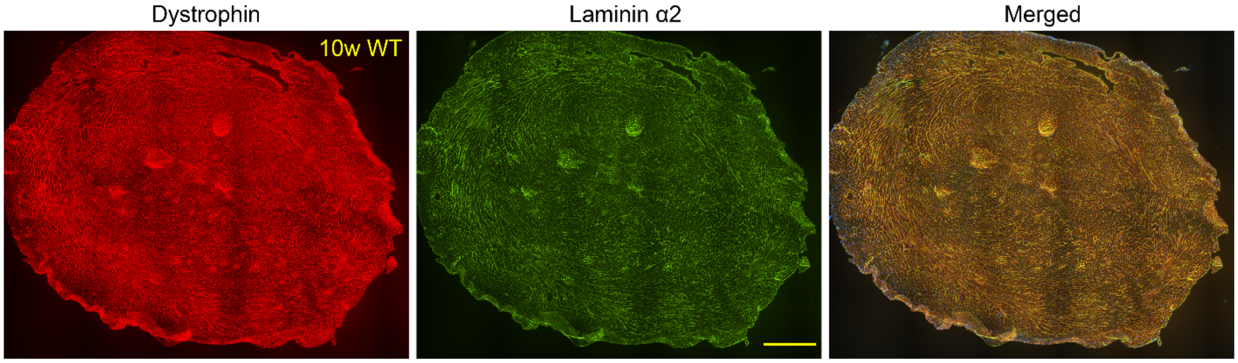

Supplementary Fig. S4. Stitched large images showing dystrophin and laminin-α2 immunostaining of the entire heart sections of a WT mouse at 10 weeks (10w) of age. Scale bars: 0.5 mm. n=3 mice.

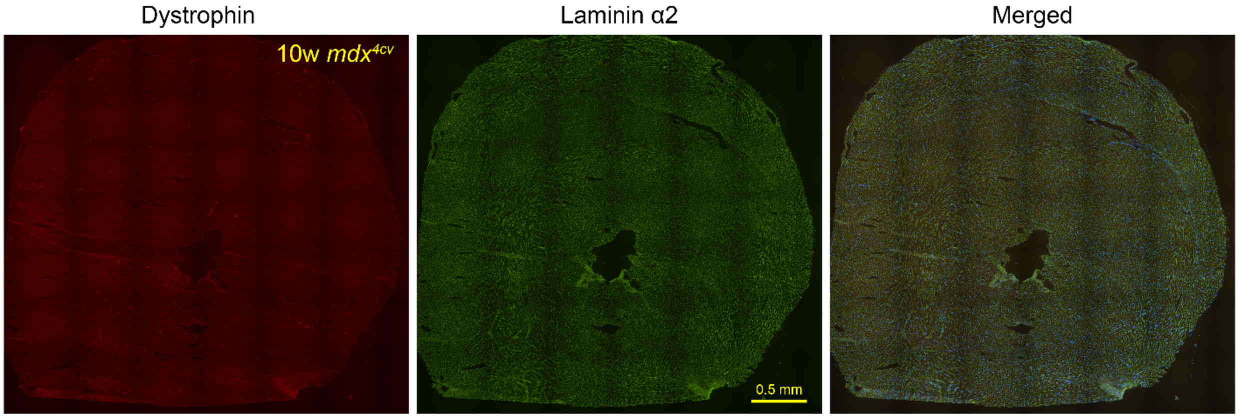

Supplementary Fig. S5. Stitched large images showing dystrophin and laminin-α2 immunostaining of the entire heart sections of a control *mdx*^{4cv} mouse at 10 weeks (10w) of age. Scale bars: 0.5 mm. n=4 mice.

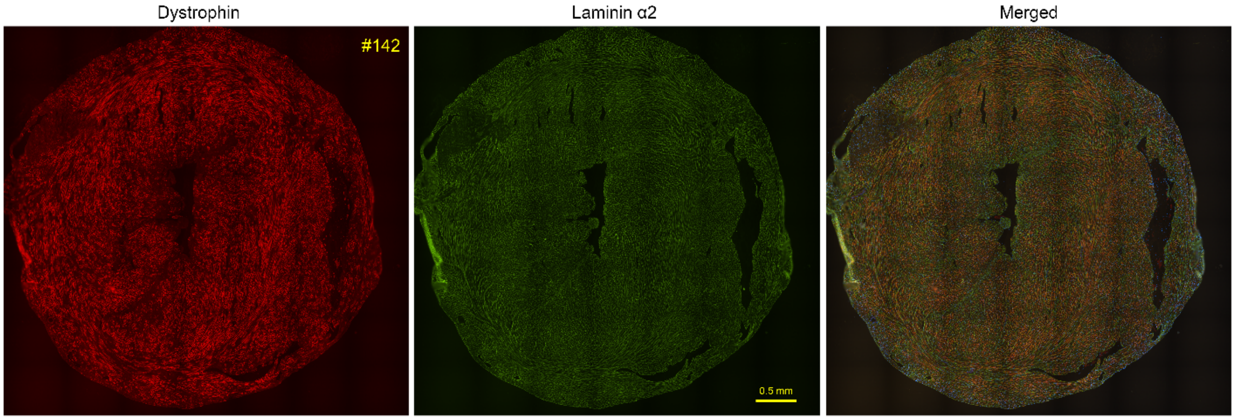

Supplementary Fig. S6. Stitched large images showing dystrophin and laminin-α2 immunostaining of the entire heart sections of an *mdx*^{4cv} mouse (mouse #142) five weeks after receiving intravenous injection of AAV9-iNG at 5 weeks of age. Scale bars: 0.5 mm. n=1 mouse.

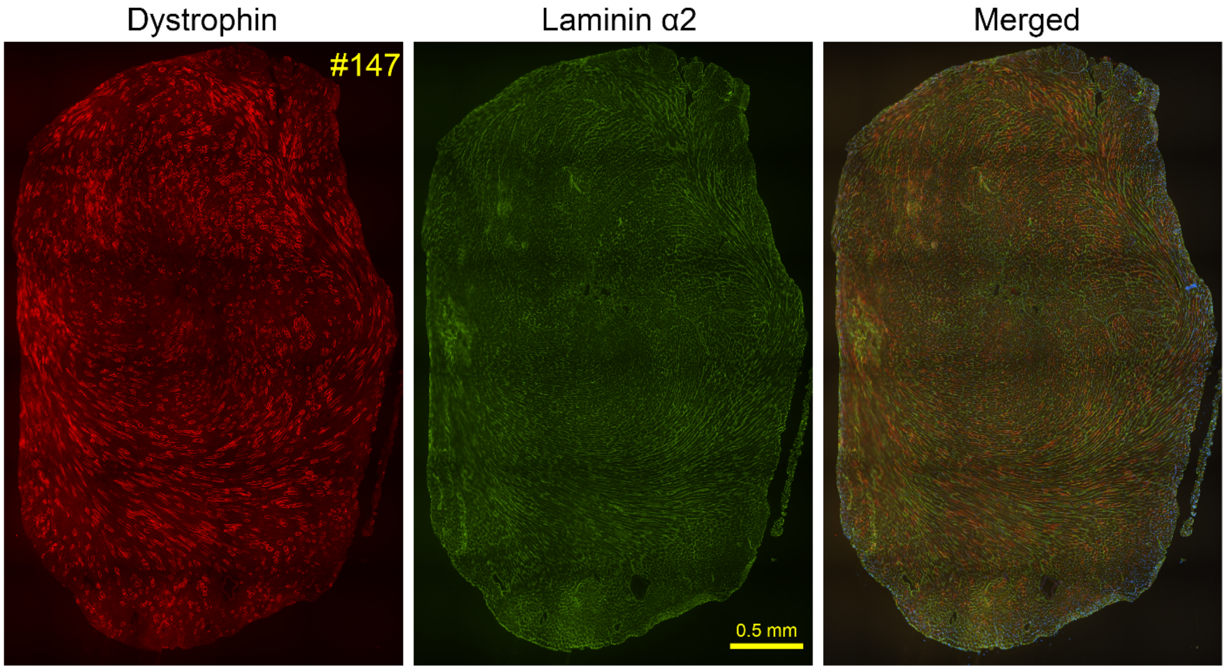

Supplementary Fig. S7. Stitched large images showing dystrophin and laminin-α2 immunostaining of the entire heart sections of an *mdx*^{4cv} mouse (mouse #147) five weeks after receiving intravenous injection of AAV9-iNG at 5 weeks of age. Scale bars: 0.5 mm. n=1 mouse.

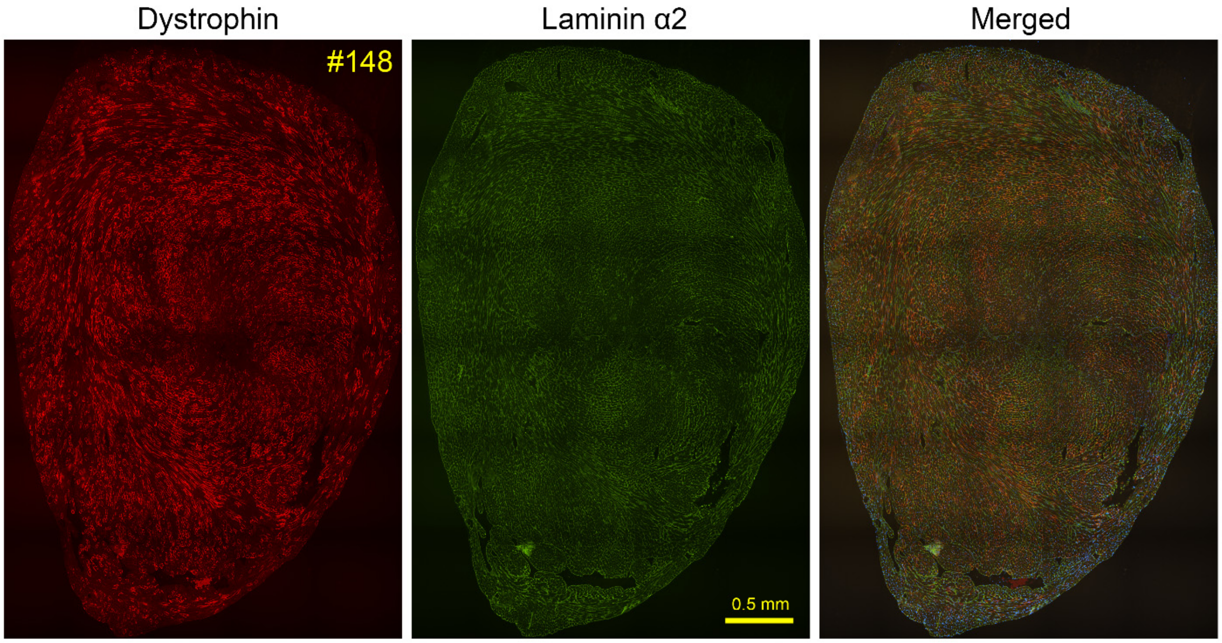

Supplementary Fig. S8. Stitched large images showing dystrophin and laminin-α2 immunostaining of the entire heart sections of an *mdx*^{4cv} mouse (mouse #148) five weeks after receiving intravenous injection of AAV9-iNG at 5 weeks of age. Scale bars: 0.5 mm. n=1 mouse.

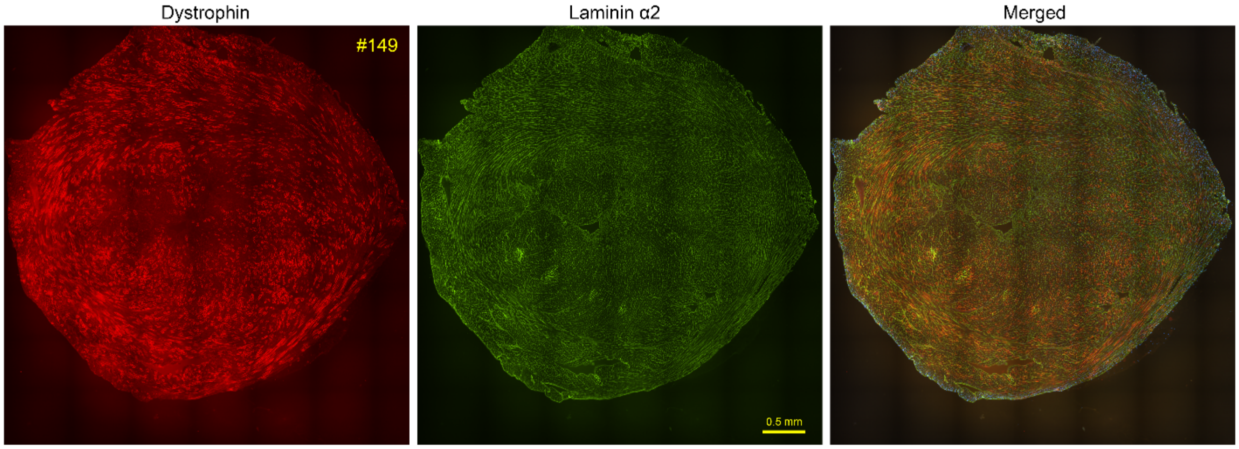

Supplementary Fig. S9. Stitched large images showing dystrophin and laminin-α2 immunostaining of the entire heart sections of an *mdx*^{4cv} mouse (mouse #149) five weeks after receiving intravenous injection of AAV9-iNG at 5 weeks of age. Scale bars: 0.5 mm. n=1 mouse.

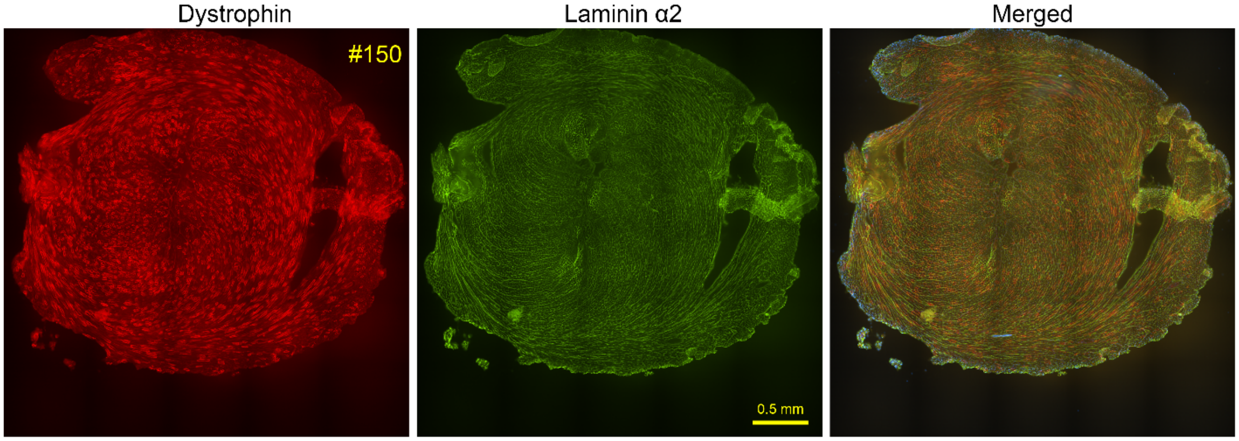

Supplementary Fig. S10. Stitched large images showing dystrophin and laminin-α2 immunostaining of the entire heart sections of an *mdx*^{4cv} mouse (mouse #150) five weeks after receiving intravenous injection of AAV9-iNG at 5 weeks of age. Scale bars: 0.5 mm. n=1 mouse.

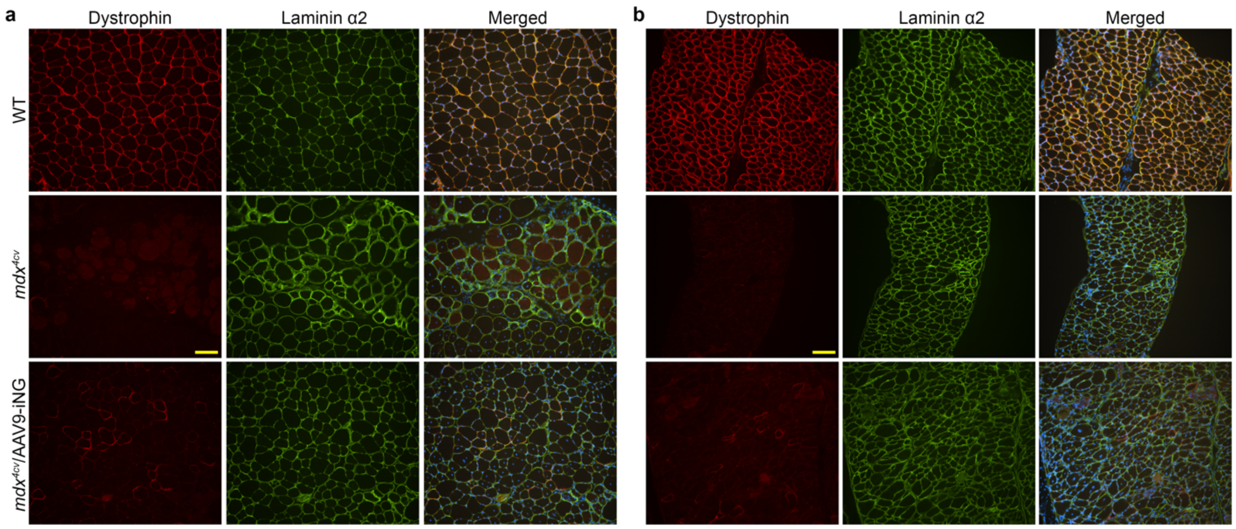

Supplementary Fig. S11. Immunofluorescence staining of dystrophin and laminin α2 in the gastrocnemius (**a**) and diaphragm (**b**) muscles from WT and *mdx*^{4cv} (10 weeks of age) treated with or without tail vein injection of AAV9-iNG. Scale bar: 100 μm. n=3 WT, 4 *mdx*^{4cv} and 5 AAV9-iNG treated *mdx*^{4cv} mice.

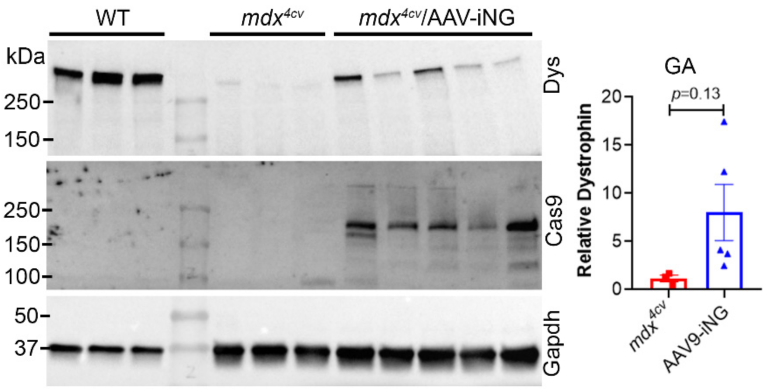

Supplementary Fig. S12. Western blot analysis of gastrocnemius muscles from WT and *mdx*^{4cv} (10 weeks of age) treated with or without tail vein injection of AAV9-iNG. n=3 *mdx*^{4cv} and 5 AAV9-iNG treated; two-tailed unpaired *t*-test. The samples were derived from the same experiment and the gels/blots were processed in parallel. Data are mean ± SD.

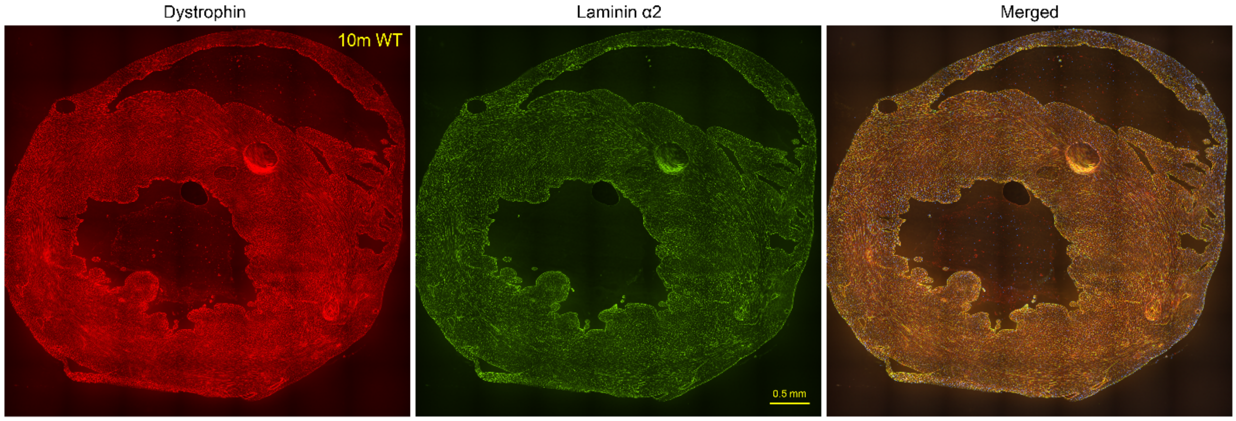

Supplementary Fig. S13. Stitched large images showing dystrophin and laminin-α2 immunostaining of the entire heart sections of a WT mouse at 10 months (10m) of age. Scale bars: 0.5 mm. n=3 mice.

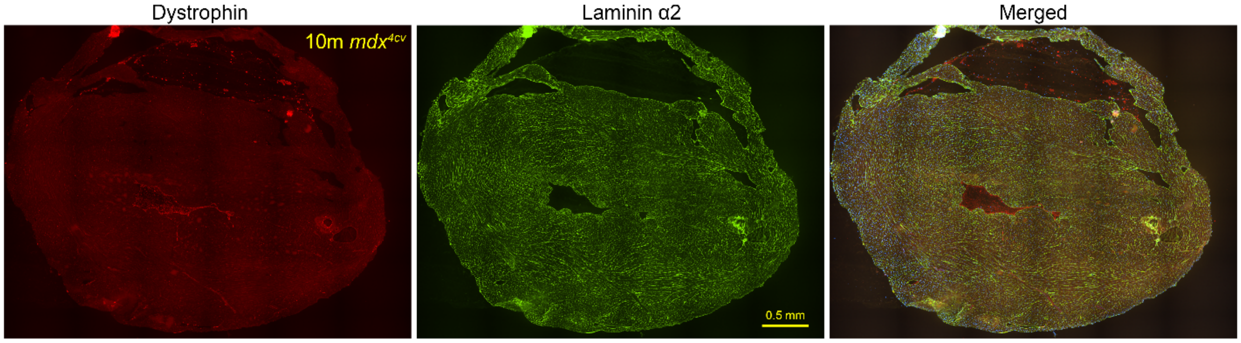

Supplementary Fig. S14. Stitched large images showing dystrophin and laminin-α2 immunostaining of the entire heart sections of a control *mdx*^{4cv} mouse at 10 months (10m) of age. Scale bars: 0.5 mm. n=3 mice.

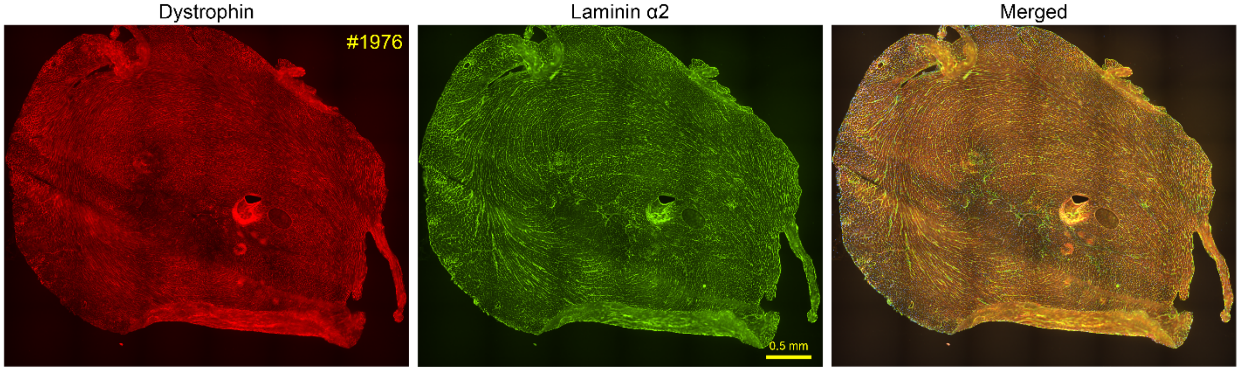

Supplementary Fig. S15. Stitched large images showing dystrophin and laminin-α2 immunostaining of the entire heart sections of an *mdx*^{4cv} mouse (mouse #1976) 9-10 months after intravenous injection of AAV9-iNG at 5 weeks of age. Scale bars: 0.5 mm. n=1 mouse.

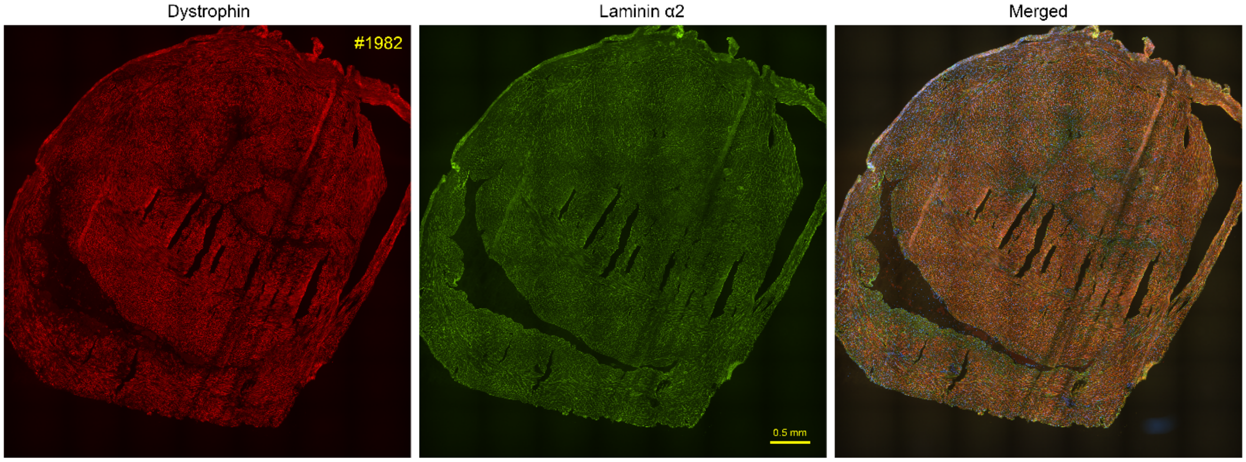

Supplementary Fig. S16. Stitched large images showing dystrophin and laminin-α2 immunostaining of the entire heart sections of an *mdx*^{4cv} mouse (mouse #1982) 9-10 months after intravenous injection of AAV9-iNG at 5 weeks of age. Scale bars: 0.5 mm. n=1 mouse.

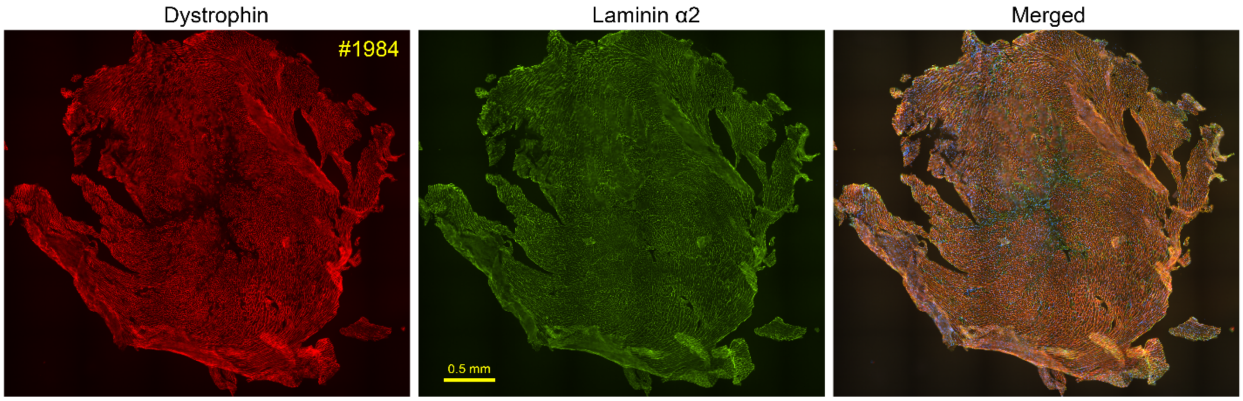

Supplementary Fig. S17. Stitched large images showing dystrophin and laminin-α2 immunostaining of the entire heart sections of an *mdx*^{4cv} mouse (mouse #1984) 9-10 months after intravenous injection of AAV9-iNG at 5 weeks of age. Scale bars: 0.5 mm. n=1 mouse.

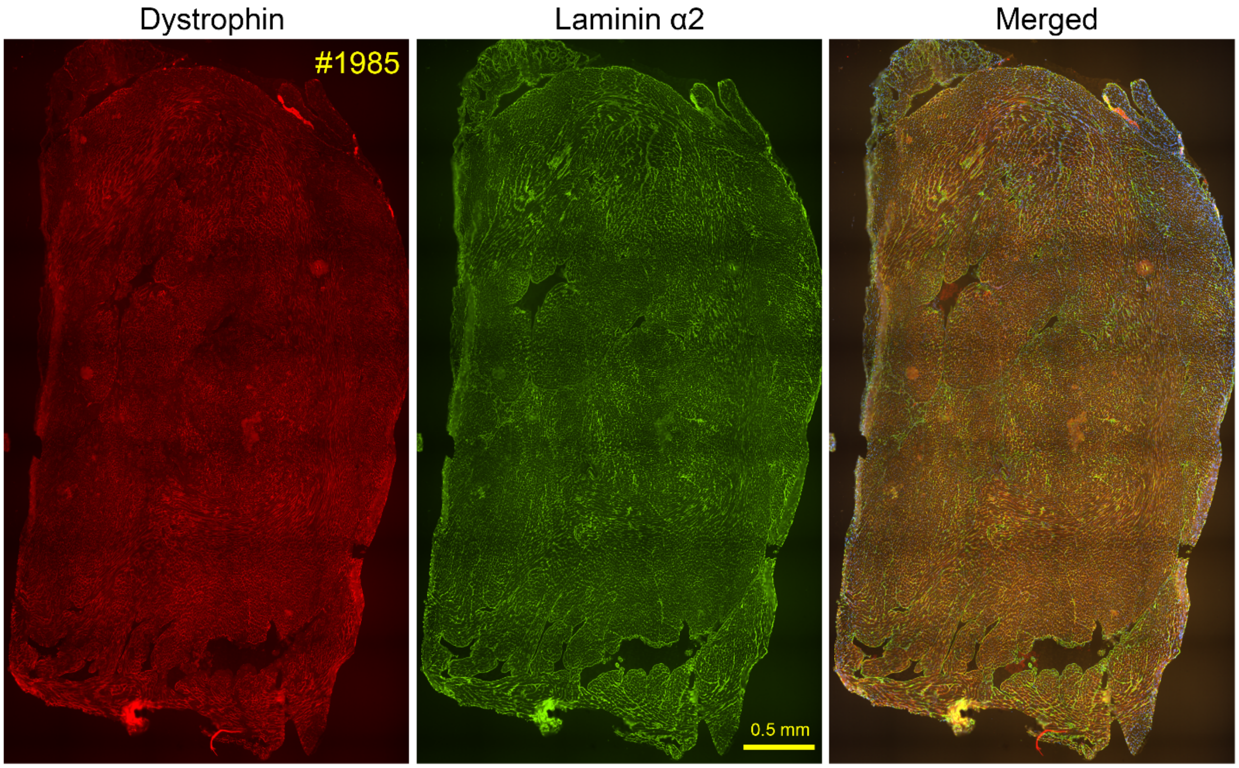

Supplementary Fig. S18. Stitched large images showing dystrophin and laminin-α2 immunostaining of the entire heart sections of an *mdx*^{4cv} mouse (mouse #1985) 9-10 months after intravenous injection of AAV9-iNG at 5 weeks of age. Scale bars: 0.5 mm. n=1 mouse.

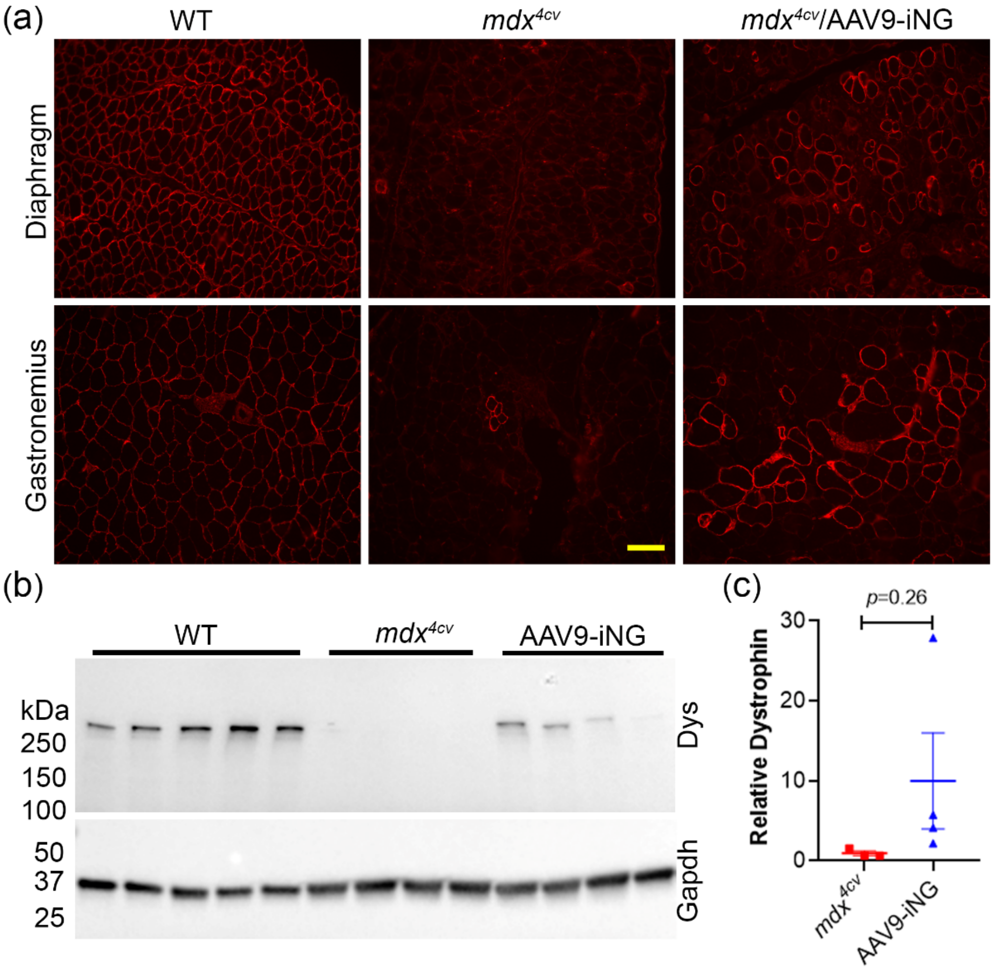

Supplementary Fig. S19. Restoration of dystrophin expression in the skeletal muscles of 10-month-old *mdx*^{4cv} mice after tail vein injection of AAV9-iNG at 5 weeks of age. (a) Immunofluorescence staining of dystrophin in diaphragm and gastrocnemius muscles of WT and *mdx*^{4cv} mice with or without systemic AAV9-iNG delivery. (b) Western blot of dystrophin expression in gastrocnemius muscles. (c) Quantification of Western blot data. n=3 *mdx*^{4cv} and 4 AAV9-iNG treated, two-tailed unpaired *t*-test. Data are mean ± s.e.m.

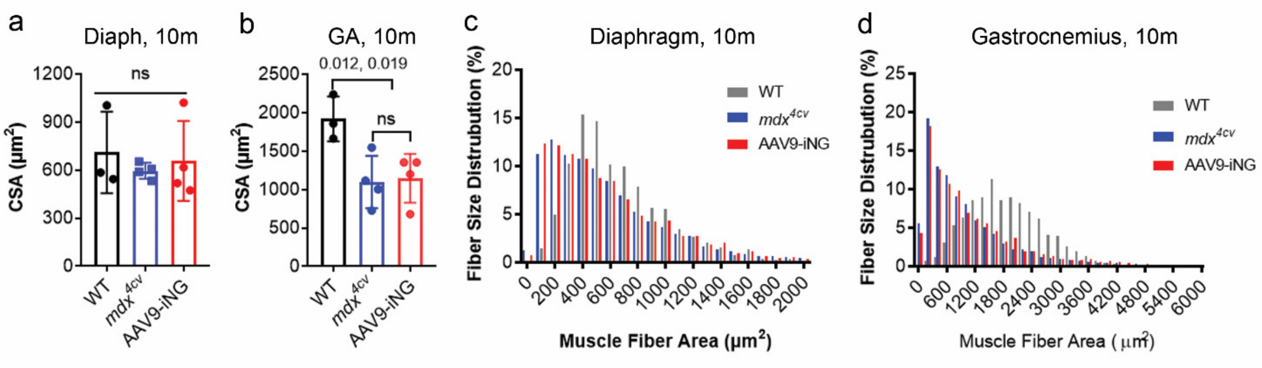

Supplementary Fig. S20. Measurement of muscle fiber size in diaphragm and gastrocnemius muscles at 10 months of age. **a**, **b**, Average CSA of diaphragm (**a**) and gastrocnemius (**b**) muscle fibers. n=3 WT, 4 *mdx*^{4cv} and 4 AAV9-iNG treated. The p values are shown on the top of the bars; ns, not significant; one-way ANOVA with Turkey's multiple comparisons test. Data are mean ± SD. **c**, **d**, Distribution of diaphragm (**c**) and gastrocnemius (**d**) muscle fiber areas.

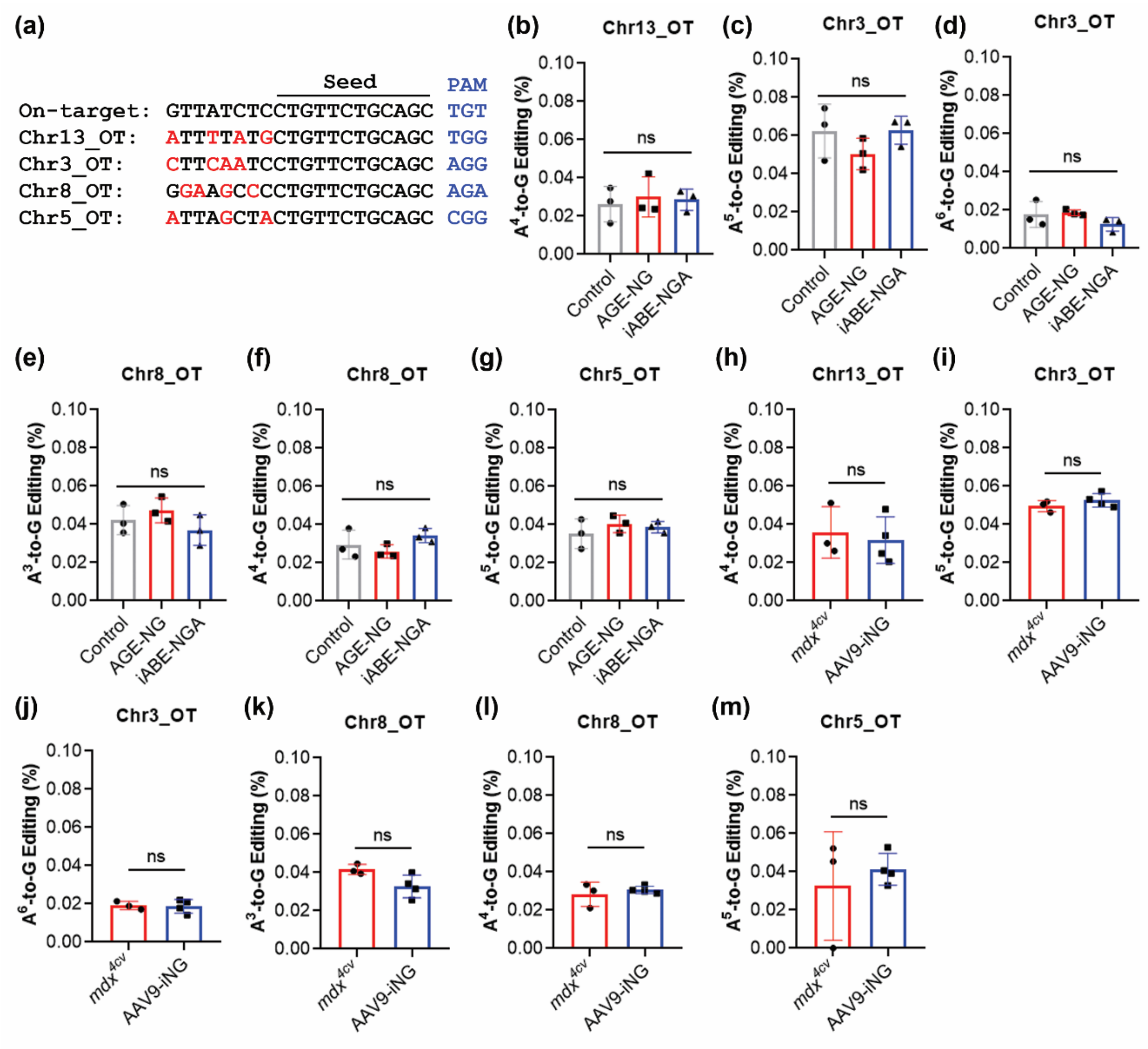

Supplementary Fig. S21. **Quantification of deep sequencing reads of the genomic DNA PCR amplicons of four additional off-target sites with the same seed region as the targeting gRNA**. (a) The off-target sequences aligned with the on-target sequence. The PAMs are shown in blue. (**b-g**) Quantification of the editing efficiencies of the target A within the editing window from Neuro-2a cells transfected with ABE-NG, iABE-NGA or control plus the gRNA. n=3 wells/group; one-way ANOVA with Turkey's multiple comparisons test. Data are mean ± SD. (**h-m**) Quantification of the editing efficiencies of the target A within the editing window from *mdx*^{4cv} mouse heart samples treated with or without AAV9-iNG. n=3 *mdx*^{4cv} and 4 AAV9-iNG treated. ns, not significant; two-tailed unpaired *t*-test. Data are mean ± SD.

PAM

		G	T	T	A^4	T	C^6	T	C	C	T	G	T	T	C	T	G	C	A	G	C	T	G	T
Mouse1	A	0.05	0.01	0.00	13.64	0.01	0.03	0.03	0.05	0.02	0.02	0.02	0.01	0.01	0.00	0.03	0.08	0.01	99.90	0.02	0.01	0.01	0.03	0.01
	C	0.00	0.03	0.07	0.01	0.03	97.93	0.04	99.86	99.96	0.09	0.00	0.02	0.03	99.96	0.11	0.00	99.96	0.00	0.00	99.97	0.07	0.00	0.02
	G	99.95	0.00	0.00	86.34	0.00	0.19	0.00	0.02	0.00	0.01	99.97	0.00	0.00	0.00	0.00	99.92	0.00	0.08	99.97	0.00	0.05	99.96	0.00
	T	0.00	99.96	99.92	0.02	99.95	1.84	99.93	0.06	0.02	99.89	0.00	99.97	99.97	0.03	99.86	0.00	0.03	0.01	0.02	0.02	99.87	0.01	99.97

		G	T	T	A^4	T	C^6	T	C	C	T	G	T	T	C	T	G	C	A	G	C	T	G	T
Mouse2	A	0.07	0.01	0.00	14.04	0.02	0.04	0.03	0.04	0.03	0.02	0.04	0.01	0.01	0.01	0.03	0.07	0.01	99.90	0.04	0.01	0.01	0.03	0.01
	C	0.00	0.03	0.06	0.00	0.02	98.27	0.04	99.90	99.92	0.09	0.00	0.02	0.03	99.97	0.14	0.00	99.94	0.01	0.00	99.97	0.09	0.00	0.02
	G	99.92	0.00	0.00	85.93	0.00	0.21	0.00	0.00	0.03	0.01	99.96	0.00	0.00	0.00	0.01	99.93	0.00	0.08	99.94	0.00	0.01	99.96	0.00
	T	0.00	99.95	99.94	0.02	99.96	1.48	99.92	0.05	0.02	99.88	0.00	99.97	99.96	0.02	99.82	0.00	0.04	0.02	0.02	0.02	99.89	0.01	99.97

		G	T	T	A^4	T	C^6	T	C	C	T	G	T	T	C	T	G	C	A	G	C	T	G	T
Mouse3	A	0.08	0.01	0.01	7.94	0.02	0.04	0.03	0.02	0.04	0.02	0.03	0.02	0.01	0.01	0.04	0.07	0.00	99.88	0.02	0.01	0.01	0.03	0.01
	C	0.00	0.06	0.06	0.01	0.05	97.74	0.07	99.97	99.94	0.09	0.00	0.02	0.03	99.98	0.15	0.00	99.95	0.01	0.00	99.96	0.09	0.00	0.02
	G	99.91	0.00	0.00	92.03	0.00	0.36	0.01	0.00	0.01	0.01	99.97	0.00	0.00	0.00	0.01	99.92	0.00	0.10	99.97	0.00	0.01	99.96	0.01
	T	0.00	99.93	99.93	0.02	99.93	1.86	99.89	0.01	0.02	99.89	0.00	99.96	99.95	0.02	99.81	0.00	0.05	0.01	0.01	0.02	99.89	0.01	99.96

		G	T	T	A^4	T	C^6	T	C	C	T	G	T	T	C	T	G	C	A	G	C	T	G	T
Mouse4	A	0.10	0.01	0.00	19.63	0.02	0.03	0.04	0.03	0.04	0.02	0.05	0.01	0.01	0.01	0.03	0.08	0.01	99.87	0.03	0.01	0.01	0.04	0.01
	C	0.00	0.04	0.06	0.01	0.02	98.51	0.06	99.93	99.94	0.10	0.00	0.03	0.04	99.97	0.17	0.00	99.93	0.01	0.00	99.96	0.12	0.00	0.02
	G	99.89	0.00	0.00	80.32	0.01	0.19	0.00	0.00	0.00	0.01	99.95	0.00	0.00	0.00	0.01	99.91	0.00	0.10	99.95	0.00	0.02	99.94	0.00
	T	0.00	99.95	99.94	0.04	99.96	1.28	99.89	0.04	0.02	99.87	0.00	99.96	99.95	0.02	99.79	0.01	0.06	0.02	0.02	0.03	99.84	0.01	99.96

Supplementary Fig. S22. Off-target activities of AAV9-iNG. The nucleotide frequency at the on-target site of the four *mdx*^{4cv} mice at 10 months after treatment with AAV9-iNG. The desired edit at A^4 is highlighted in green and the bystander C^6 edit in red.

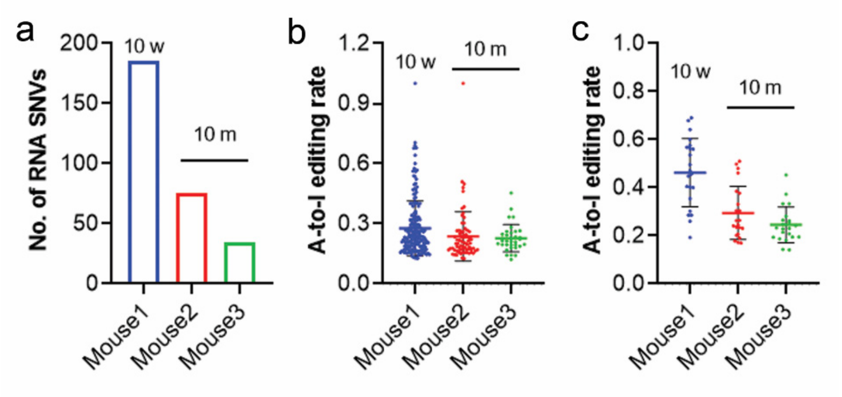

Supplementary Fig. S23. Off-target RNA editing activities in the *mdx*^{4cv} mouse hearts (10 weeks [10 w] and 10 months [10 m] of age) induced by systemic delivery of AAV9-iNG. **a**, The number of RNA SNVs in three AAV9-iNG treated mouse hearts after filtering the RNA SNVs in three control mouse hearts. **b**, The A-to-I editing rate of all RNA SNVs. Data are mean ± SD. N=3 mice. **c**, The A-to-I editing rate of the common RNA SNVs in the three AAV9-iNG treated mouse hearts. Data are mean ± SD. n=3 mice.

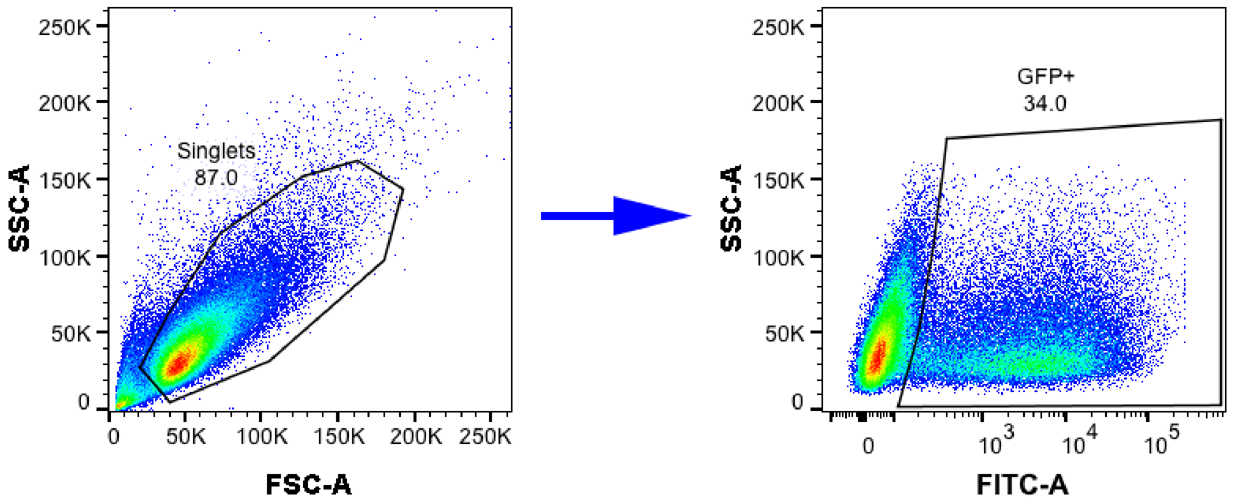

Supplementary Fig. S24. Flow cytometry gating trees. The gating strategy is used for both Figure 1d and Figure 4d.
