# Supplementary material for: Efficient precise in vivo base editing in adult dystrophic mice
Source: Nat Commun. 2021 Jun 17;12:3719. doi: 10.1038/s41467-021-23996-y (PMC8211797; doi:10.1038/s41467-021-23996-y)
Supplement: Supplementary file 2 — Reporting Summary [file 41467_2021_23996_MOESM2_ESM.pdf]

nature research

Corresponding author(s): Renzhi Han

Last updated by author(s): Apr 5, 2021

Reporting Summary

Nature Research wishes to improve the reproducibility of the work that we publish. This form provides structure for consistency and transparency in reporting. For further information on Nature Research policies, see Authors & Referees and the Editorial Policy Checklist .

Statistics

For all statistical analyses, confirm that the following items are present in the figure legend, table legend, main text, or Methods section.

n/a	Confirmed	
☐	☒	The exact sample size (*n*) for each experimental group/condition, given as a discrete number and unit of measurement
☐	☒	A statement on whether measurements were taken from distinct samples or whether the same sample was measured repeatedly
☐	☒	The statistical test(s) used AND whether they are one- or two-sided<br>*Only common tests should be described solely by name; describe more complex techniques in the Methods section.*
☒	☐	A description of all covariates tested
☐	☒	A description of any assumptions or corrections, such as tests of normality and adjustment for multiple comparisons
☐	☒	A full description of the statistical parameters including central tendency (e.g. means) or other basic estimates (e.g. regression coefficient) AND variation (e.g. standard deviation) or associated estimates of uncertainty (e.g. confidence intervals)
☐	☒	For null hypothesis testing, the test statistic (e.g. *F*, *t*, *r*) with confidence intervals, effect sizes, degrees of freedom and *P* value noted<br>*Give P values as exact values whenever suitable.*
☒	☐	For Bayesian analysis, information on the choice of priors and Markov chain Monte Carlo settings
☒	☐	For hierarchical and complex designs, identification of the appropriate level for tests and full reporting of outcomes
☒	☐	Estimates of effect sizes (e.g. Cohen's *d*, Pearson's *r*), indicating how they were calculated

Our web collection on statistics for biologists contains articles on many of the points above.

Software and code

Policy information about availability of computer code

Data collection	NIS-Elements AR version 4.50 was used to collect fluorescence images; ChemiDoc XRS+ system was used to collect Western blot data; DMA v5.501 was used to collect muscle contractility data; Flow cytometry was carried out on Becton Dickinson LSR II with BD FACSDiva software (version 8.0.1).
Data analysis	Graphpad prism 8.0.1 was used to analyze the data. FlowJo 10.4 was used to analyze FACS data collected with the BD FACSDiva software (version 8.0.1). Adobe Photoshop 2020 was used to assemble figures. Western blots were imaged and quantified using Image Lab 6.0.1 software. Sanger sequencing trace data were analyzed by using BEAT v1.0, which is published and available at https://github.com/HanLab-OSU/Beat. Next generation sequencing data were analyzed by using CRISPResso2. RNAseq data were analyzed by following the GATK Best Practices for RNA-seq variant calling with the following softwares: STAR version 1.5.2, Picard tools version 2.19.0, SplitNCigarReads, IndelRealigner, BaseRecalibrator and HaplotypeCaller tools from GATK version 4.1.2.0.

For manuscripts utilizing custom algorithms or software that are central to the research but not yet described in published literature, software must be made available to editors/reviewers. We strongly encourage code deposition in a community repository (e.g. GitHub). See the Nature Research guidelines for submitting code & software for further information.

Data

Policy information about availability of data

All manuscripts must include a data availability statement. This statement should provide the following information, where applicable:

- Accession codes, unique identifiers, or web links for publicly available datasets
- A list of figures that have associated raw data
- A description of any restrictions on data availability

The sequencing data have been deposited in the NCBI SRA under project accession numbers (PRJNA673243). All relevant data supporting the key findings of this study are available within the article and its Supplementary Information files or from the corresponding author upon reasonable request. Source data are provided with this paper.

Field-specific reporting

Please select the one below that is the best fit for your research. If you are not sure, read the appropriate sections before making your selection.

- [x] Life sciences
- [ ] Behavioural & social sciences
- [ ] Ecological, evolutionary & environmental sciences

For a reference copy of the document with all sections, see nature.com/documents/nr-reporting-summary-flat.pdf

Life sciences study design

All studies must disclose on these points even when the disclosure is negative.

Sample size	Sample size is estimated with G-power software 3.1 by the significance level (0.05), effect size (based on the data in our previous studies) and a given power (usually 0.8). We also conducted power analysis after the experiment and found enough power value (>0.8) in each statistical analysis.
Data exclusions	No data exclusion
Replication	All attempts for replication were successful. A minimum of three independent experiments (or animals) were included for all FACS, ELISA, immunofluorescence staining, Western blot and contractile measurements.
Randomization	Mice (only males as DMD primarily affects boys) and cells were assigned randomly into experimental groups and processed in an arbitrary order.
Blinding	All the enrolled mice or subsequent samples were labeled only with mouse ID numbers without genotype or type of treatment information. Genotype or treatment type were decoded after the data acquisition and quantification analysis were complete. The culture studies (transfection, DNA/RNA isolation, PCR, sequencing, FACS) were carried out identically through standard procedures that should not bias outcomes.

Reporting for specific materials, systems and methods

We require information from authors about some types of materials, experimental systems and methods used in many studies. Here, indicate whether each material, system or method listed is relevant to your study. If you are not sure if a list item applies to your research, read the appropriate section before selecting a response.

Materials & experimental systems

n/a	Involved in the study	
☐	☒	Antibodies
☐	☒	Eukaryotic cell lines
☒	☐	Palaeontology
☐	☒	Animals and other organisms
☒	☐	Human research participants
☒	☐	Clinical data

Methods

n/a	Involved in the study	
☒	☐	ChIP-seq
☐	☒	Flow cytometry
☒	☐	MRI-based neuroimaging

Antibodies

Antibodies used	For ELISA: AAV2 (A20, 1:1000 to 1:1E9, cat. # 03-65155, American Research Products Inc, Waltham, MA) and anti-SpCas9 antibody (C15310258, 1:1000 to 1:1E9, Diagenode, Denville, NJ); For immunofluorescence staining: antibodies against dystrophin (ab15277, 1:100, Abcam, Cambridge, MA) and laminin-α2 (ALX804-190-C100, 1:100, Enzo Life Sciences Inc, Farmingdale, NY). Secondary antibodies include Alexa Fluor 488 goat anti-rat IgG (A-11006, 1:400, Invitrogen, Carlsbad, CA) and Alexa Fluor 568 donkey anti-rabbit IgG (A10042, 1:400, Invitrogen, Carlsbad, CA); For Western blot: The rabbit polyclonal anti-dystrophin (E2660, 1:500, Spring Bioscience, Pleasanton, CA), rabbit polyclonal anti-Cas9 (C15310258, 1:1000, Diagenode, Denville, NJ) and rabbit monoclonal anti-Gapdh (2118S, 14C10, 1:2000, Cell Signaling Technology, Danvers, MA) ; HRP conjugated goat anti-mouse (7076S, 1:4000, Cell Signaling Technology, Danvers, MA) and goat anti-rabbit (7074S, 1:4000, Cell Signaling Technology, Danvers, MA)
Validation	All antibodies have been tested for reactivity against the appropriate species on the specification sheets on the providers' websites or in published articles. The dystrophin antibody (ab15277, 1:100, Abcam, Cambridge, MA) used for immunofluorescence staining detected sarcolemmal dystrophin in wild-type muscles but not in mdx4cv muscles. The dystrophin antibody (E2660, 1:500, Spring Bioscience, Pleasanton, CA) used for Western blot detected bands at the expected molecular weight in WT mice but not in mdx4cv mice. The laminin-α2 antibody (ALX804-190-C100, 1:100, Enzo Life Sciences Inc, Farmingdale, NY) recognizes an N-terminal portion of the α2 chain that is deleted in congenital muscular dystrophies and reacts with human and mouse. According to the respective datasheet or the manufacturer's website, the anti-SpCas9 antibody C15310258, Diagenode, Denville, NJ) was raised against Cas9 from Streptococcus pyogenes, and validated for Western blotting,

immunoprecipitation, immunofluorescence and CHIP assays; the rabbit monoclonal anti-Gapdh antibody (2118S, 14C10, 1:2000, Cell Signaling Technology, Danvers, MA) reacts with human, mouse, rat, monkey, bovine, and pig GAPDH, and works on Western blotting, immunofluorescence, and flow cytometry; the AAV2 (A20, cat. # 03-65155, American Research Products Inc, Waltham, MA) antibody recognizes a conformational epitope of assembled AAV2 capsids, not present in denatured and native unassembled capsid proteins.

Eukaryotic cell lines

Policy information about cell lines

Cell line source(s)	HEK293 and Neuro-2a cells were from ATCC.
Authentication	The HEK293 and Neuro-2a cells were not authenticated.
Mycoplasma contamination	The HEK293 and Neuro-2a cells were not tested for mycoplasma contamination.
Commonly misidentified lines (See ICLAC register)	To the best of our knowledge no misidentified cell lines have been used in this study.

Animals and other organisms

Policy information about studies involving animals; ARRIVE guidelines recommended for reporting animal research

Laboratory animals	C57BL/6J and mdx4cv (B6Ros.Cg-Dmdmdx-4Cv/J) were purchased from the Jackson Laboratory. Only males at the age of 5 weeks to 10 months were used in this study.
Wild animals	The study did not involve wild animals.
Field-collected samples	The study did not involve samples collected from the field.
Ethics oversight	The animal experiments were ethically reviewed by the Animal Care, Use, and Review Committee of the Ohio State University and carried out in accordance with animal use guidelines.

Note that full information on the approval of the study protocol must also be provided in the manuscript.

Flow Cytometry

Plots

Confirm that:

- [x] The axis labels state the marker and fluorochrome used (e.g. CD4-FITC).
- [x] The axis scales are clearly visible. Include numbers along axes only for bottom left plot of group (a 'group' is an analysis of identical markers).
- [x] All plots are contour plots with outliers or pseudocolor plots.
- [x] A numerical value for number of cells or percentage (with statistics) is provided.

Methodology

Sample preparation	At 72 hours post transfection, cultured cells in 6-well plates were trypsinized, centrifuged, resuspended in MACS buffer, filtered through 35µm Nylon mesh to obtain single-cell suspension.
Instrument	Becton Dickinson LSR II.
Software	BD FACSDiva v8.0.1
Cell population abundance	No cells were sorted from cell population and no post-sort analysis was done. Cell clumps and debris were excluded.
Gating strategy	Gating for edited cell analyses started as singlet (SSC-A vs. FSC-A) and then GFP+.

- [x] Tick this box to confirm that a figure exemplifying the gating strategy is provided in the Supplementary Information.
